# On the spontaneous stochastic dynamics of a single gene: complexity of the molecular interplay at the promoter

**DOI:** 10.1186/1752-0509-4-2

**Published:** 2010-01-08

**Authors:** Antoine Coulon, Olivier Gandrillon, Guillaume Beslon

**Affiliations:** 1 Université de Lyon, Université Lyon 1, Centre de Génétique Moléculaire et Cellulaire (CGMC), CNRS UMR5534, F-69622 Lyon, France; 2 Université de Lyon, INSA-Lyon, Laboratoire d'InfoRmatique en Image et Systemes d'information (LIRIS), CNRS UMR5205, F-69621 Lyon, France; 3 Rhône-Alpes Complex Systems Institute (IXXI), F-69007 Lyon, France

## Abstract

**Background:**

Gene promoters can be in various epigenetic states and undergo interactions with many molecules in a highly transient, probabilistic and combinatorial way, resulting in a complex global dynamics as observed experimentally. However, models of stochastic gene expression commonly consider promoter activity as a two-state on/off system. We consider here a model of single-gene stochastic expression that can represent arbitrary prokaryotic or eukaryotic promoters, based on the combinatorial interplay between molecules and epigenetic factors, including energy-dependent remodeling and enzymatic activities.

**Results:**

We show that, considering the mere molecular interplay at the promoter, a single-gene can demonstrate an elaborate spontaneous stochastic activity (eg. multi-periodic multi-relaxation dynamics), similar to what is known to occur at the gene-network level. Characterizing this generic model with indicators of dynamic and steady-state properties (including power spectra and distributions), we reveal the potential activity of any promoter and its influence on gene expression. In particular, we can reproduce, based on biologically relevant mechanisms, the strongly periodic patterns of promoter occupancy by transcription factors (TF) and chromatin remodeling as observed experimentally on eukaryotic promoters. Moreover, we link several of its characteristics to properties of the underlying biochemical system. The model can also be used to identify behaviors of interest (eg. stochasticity induced by high TF concentration) on minimal systems and to test their relevance in larger and more realistic systems. We finally show that TF concentrations can regulate many aspects of the stochastic activity with a considerable flexibility and complexity.

**Conclusions:**

This tight promoter-mediated control of stochasticity may constitute a powerful asset for the cell. Remarkably, a strongly periodic activity that demonstrates a complex TF concentration-dependent control is obtained when molecular interactions have typical characteristics observed on eukaryotic promoters (high mobility, functional redundancy, many alternate states/pathways). We also show that this regime results in a direct and indirect energetic cost. Finally, this model can constitute a framework for unifying various experimental approaches. Collectively, our results show that a gene - the basic building block of complex regulatory networks - can itself demonstrate a significantly complex behavior.

## Background

Considered for a long time to be insignificant variations around a significant mean, stochasticity in gene expression is now clearly demonstrated to be important in many situations and in many organisms [1-16] and to participate in various biological processes [15-20], as formerly proposed [21]. The molecular bases of this stochasticity are multiple and constitute now a major subject of investigation. They are frequently distinguished between intrinsic and extrinsic stochasticity [1,22]. Although this distinction requires a clear statement of the considered system [23], this system is often (eg. as in [1]) implicit and corresponds to what we would call a "node" in a regulatory network. Then, extrinsic and intrinsic stochasticity are respectively the *propagation through *this node of global fluctuations of the concentration of transcription factors (TFs), RNA polymerase ..., and the *generation *of randomness due to the molecular events, discrete and probabilistic in nature, that take place within the system (TFs binding to the promoter, transcription initiation, RNA degradation ...).

A major challenge in this field is to isolate and characterize the various sources of stochasticity in different organisms (from prokaryotes to higher eukaryotes) by theoretical [22-44] and/or experimental means [1-12]. Stochasticity gets significant when some discrete molecular events become rare. Typically, when RNA or proteins are in low copy number, synthesis and degradation events are rare and represent important variations relatively to the total amount of these molecular species. This generates a so-called Poisson noise at both RNA and protein levels [23]. Also, because at low copy number TFs cannot be considered to be uniformly distributed, spatial proximity of the few TFs to the promoter becomes important and contributes to enhance stochasticity [33]. Finally, and independently of molecule concentrations, the transition of the promoter between different states (chromatin state, presence/absence of a TF ...) provokes heterogeneity in transcription (eg. bursts [3,4,6,14,15]) and appears to be a major source of stochasticity [5].

The vast literature describing the molecular machinery involved at the promoter reports very elaborate properties. The various classes of molecules involved in transcriptional regulation show quite wide-ranging but surprisingly short residence time (typically few seconds) within complexes [45-48]. Moreover, despite this rapid turnover of most (if not all) molecules, eukaryotic regulatory complexes also demonstrate a clear slow-timescale activity: They proceed through a periodic pattern of occupancy level by molecules, conformal changes and epigenetic modifications with a precise timing and a period in the order of few tens of minutes [48-52]. This phenomenon referred to as the *cyclical recruitment*, *occupancy pattern *or *loading profile *of molecules on the promoter, or even as *chromatin breathing*. This provides a new vision of regulatory complexes as highly dynamic structures in constant assembly and disassembly, with alternative functionally redundant pathways of formation and with phenomena occurring concomitantly at different timescales [48,52-55]. An important point is that, in both prokaryotes [56,57] and eukaryotes [58-61], the association/dissociation of most molecules involves cooperation and competition with the other molecules bound to the promoter. Also, alternative conformations (DNA looping, chromatin open/closed state, nucleosome position along DNA ...), post-translational covalent modifications of histone tails residues (acetylation, methylation, phosphorylation ... defining the "histone code" [62]) and DNA methylation are other factors that influence and are influenced by the molecules present on the promoter in a dynamic, highly combinatorial and possibly energy-dependent manner [53,60-66]. These combinatorial aspects also take place in RNA polymerase recruitment [49,50,66] and provides the promoter with a variety of levels of transcriptional competency [48,56,61,65], far from the binary vision of all-or-nothing active/inactive genes. All this elaborate molecular interplay provides the regulatory structure with a complex dynamics and certainly have major outcomes on stochasticity of gene expression. Two-state *on/off *promoters with exponential switching times [3,4,14,23-27,30,32,34-37,40] (or slightly more detailed models [2,33,44]) have been used for a long time and gave many valuable insights into the importance of promoter dynamics. Beyond this simple description, few authors recently focused on more precise descriptions of promoters from the viewpoint of stochastic gene expression [41-43,67,68]. These studies revealed interesting results but remained focused on specific features (eg. non-exponential waiting times, shape of regulatory input function) or on restricted systems (eg. prokaryotic energetically-closed systems) so that general principles on the capacity of regulatory molecular interplay remain mostly unexplored. Recently, the stochastic dynamics of a eukaryotic promoter has been modeled considering the interplay between TFs and chromatin modifications and in relation with experimental data [52]. This work provided several important insights into the relation between single-cell and population dynamics and showed that the approach is very promising. However, being essentially based on simulations, the understanding of the structure of the dynamics and all its potentiality as well as parameter explorations remain more limited than when using an analytical approach. Moreover, the very synthetic metrics commonly used in studies of stochastic gene expression (eg. variance normalized by square mean) miss most of both steady-state and dynamic aspects of the system's activity. Here, we employ more comprehensive measures such as power spectra or autocorrelations [7,8,12,29,30,33,34,69,70] and full distributions [2,6,9,27,28,35-37,40] that are known to reveal many more relevant features. A power spectrum is a measure that describes the temporal fluctuations of a signal (eg. a protein level) by revealing its frequency content. For instance, a peak in a power spectrum at a given frequency tells that the considered signal tends to repeat itself periodically (the sharper the peak, the more precisely the signal is repeated). On the opposite, a plateau up to a given frequency followed by a decrease tells that the signal fluctuates aperiodically with a typical correlation time as short as the plateau spans over high frequencies.

We first present a generic promoter-centered model of the stochastic expression of a single gene - that can represent arbitrary regulatory systems from prokaryotes to eukaryotes - and describe its spontaneous activity in terms of power spectra, normalized variance and full distribution. It reveals that a single gene can demonstrate the same type of complex dynamics as those that were previously identified at the network-level (eg. multi-periodic multi-relaxation dynamics). In particular, it can reproduce with realistic parameters the periodic occupancy patterns observed experimentally on eukaryotic promoters and highlight the central role of energy-dependence in this context. Then we show that instantiation into minimalist systems can help to identify novel properties (eg. stochasticity induced at high TF concentration) and verify their applicability to larger and more biologically plausible systems. Finally, we show how TF concentration can modulate many aspects of the promoter activity in a highly complex and flexible manner, suggesting the transcriptional regulation as a central piece for the cell to control and take advantage of stochasticity. We discuss our results and their theoretical and experimental implications in different fields.

## Methods

**Figure 1:**
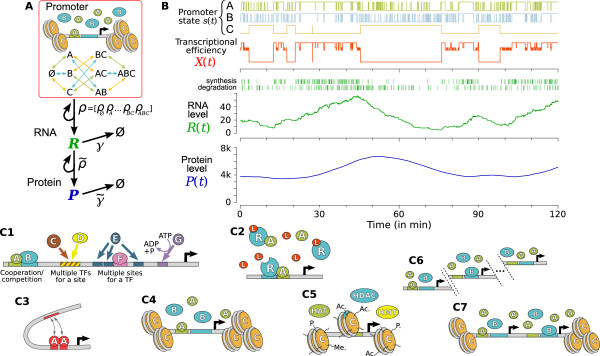
**Promoter-centered model of gene expression**. **(A) **All the complex molecular interplay between an arbitrary number of TFs is described generically while the subsequent steps of gene expression are kept simple but explicit. **(B) **Promoter state fluctuations determine the time-dependent transcriptional efficiency *X*(*t*) that propagates successively to RNA level *R*(*t*) and protein level *P*(*t*) through coupled stochastic synthesis/degradation processes. In this example with realistic timescales and parameter values (cf table S1 of Additional file 1 for a complete description), TFs *A *and *B *cooperate and the closed state of chromatin *C *compete with their association. The highest and lowest transcription rates correspond respectively to open chromatin with *A *and *B *bound to the promoter and closed chromatin. **(C) **This model can represent many different aspects of regulation (see *Description ability*) making it relevant for describing either prokaryotic or eukaryotic systems.

Dedicated to the study of the impact of stochastic promoter dynamics on gene expression, this model (figures 1A and 1C) describes the molecular events of various sorts occurring at the promoter. Mechanisms subsequent to transcription initiation are kept simple (as in most models) but explicit so that we can assess how promoter stochasticity propagates up to RNA and protein levels and confronts to other sources of gene-intrinsic stochasticity. The reader can refer to the Additional file 1 for an extensive description of the model. For simplicity, we only give here a brief description of a simplified version. However, all derivation and conclusions of this paper stand for both versions. The following model shares some similarities with previous models [42,52,67,68,71-73] and can be solved as an instance of generic techniques [23,34,69,70,74,75] or simulated using generic frameworks [76-78]. We highlight here the key difference in its definition, interpretation and resolution.

### Molecular interplay at the promoter - Kinetic formulation

We first consider TF molecules associating with and dissociating from the promoter. As we will see, these can actually represent many other aspects of regulatory complexes. We consider an arbitrary number *N *of TFs, noted *f* ∈ ℱ (eg. ℱ = {*A*, *B*, *C*, ...}). The set of TFs that are bound to the promoter at a given instant (2^*N *^possible combinations) is referred to as the promoter state and noted *s *∈ {ø, *A*, *B*, *AB*, *C*, *AC*, ...}. Classically, a TF *A *at concentration [*A*] binds and unbinds on/from its target site with rates [*A*] *k*_on _and *k*_off _respectively. However, because of cooperation and competition [56-61], the association and dissociation constants *k*_on _and *k*_off _of any TF actually depend on the combination of all the other TFs present on the promoter. We define the *N *× 2^*N *^matrix **k**^0 ^summarizing the association and dissociation constants of each of the *N *TFs and for each of the 2^*N *^states.  describes the transition from state *s *to state *s *⊖ *f *(where ⊖ denotes the symmetric difference between sets; eg. *ABC *⊖ *B *= *AC *and *AC *⊖ *B *= *ABC*). Multiplying each association rate by the concentration [*f*] of the TF that binds, we obtain the *N *× 2^*N *^matrix **k **describing all the transition rates of the weighted directed graph of promoter states (figure 1A). To focus on gene-intrinsic stochasticity, any source of gene-extrinsic stochasticity is avoided by considering TFs to be uniformly distributed in space and in constant concentration, so that the *N*-vector [*f*]_*f *∈ ℱ _of TFs' concentrations is a parameter of the model. This generic description can represent arbitrarily complex relations of combinatorial cooperation/competition and kinetic influence.

Although for simplicity this short description of the model as well as the examples in this paper do not consider TFs associating and dissociating simultaneously as a complex (eg. ∅ ⇄ *AB*), the model can actually account for these transitions (cf Additional file 1, §2). The influence of considering such reactions is discussed (cf *Discussion*).

Combinatorial cooperation/competition also takes place in RNA polymerase recruitment and provide each promoter state with a certain competency to initiate transcription [48-50,56,61,65,66]. This is described more accurately than the binary view of *on*/*off *promoter activity, by a 2^*N*^-vector ***ρ ***of state dependent transcriptional efficiency.

### Subsequent steps of gene expression

As in most models of stochastic gene expression, RNA and protein levels (noted *R*(*t*) and *P*(*t*) respectively) follow classical stochastic birth-and-death processes with instantaneous and first order reactions (cf figure 1A). The time-dependent transcriptional efficiency of promoter *X*(*t*) determines the synthesis rate of RNA molecules which, in turn, degrades with rate *γ*. Protein level *P*(*t*) is driven by the translation of RNA molecules at rate  and a degradation rate  (figure 1B). Implications of the very usual simplifications of instantaneous transcription and translation and first order degradations will be discussed (see *Discussion*).

### Energetic signification

Regulation is classically approached with thermodynamic methods [79,80]. We show that our model can be expressed in energetic terms and constitute a generalization of these approaches by extending the range of systems that can be represented (ie. including energy-consuming systems such as eukaryotic promoters) and the type of metrics that can predicted (ie. including measures of dynamic and stochastic properties). The usual thermodynamic formulation of cooperative and competitive association/dissociation of TFs [67,68,79,80] is equivalent to assign a Gibbs free energy to each promoter state. For our system, it corresponds to a 2^*N*^-vector **G^0 ^**in the standard condition (ie. all TFs having unit concentration. For arbitrary concentrations, *G*_*s *_=  + k_B_*TΣ*_*f *∉ *s *_log [*f*]. *T*, temperature; k_B_, Boltzmann constant). This representation allows one to predict the equilibrium steady-states (by applying a Boltzmann factor) and has been widely used to investigate the mean aspects of prokaryotic regulation [79,80]. But it has the drawback to restrict the analysis to energetically-closed systems and, not carrying any kinetic information, it forbid any investigation of the stochastic aspects of expression.

For this energetic formulation to be equivalent to the kinetic one, we have to consider an additional set of energy values that are difficult to access experimentally [68], namely the energy of the activation barrier for each reaction. Representing them by the *N *× 2^*N *^matrix **E^0^**, the energy that must be overcome for reaction *s* → *s* ⊖ *f* to occur is  - . The kinetic constants **k**^0 ^can then be obtained as . This reformulation allows us to explicitly make the distinction between open and closed systems (ie. involving or not energy-dependent reactions). For a closed system, nothing else than TFs and promoter DNA are involved and the energy of the activation barrier is the same in both directions of each reaction *s* ⇄ *s* ⊖ *f* so that . For an open system, energy-dependent reactions (eg. involving ATP hydrolysis) are possible, resulting in  (the difference being the energy received by the system). It can be shown that it is only in the case of an open system that the transition graph (figure 1A) can contain directed cycles so that the detailed balance property of the chemical system (corresponding to the reversibility property of the underlying Markov chain) does not hold [75] (cf Additional file 1, §2.2). This property has meaningful biological implications in the context of promoter dynamics and is most likely an essential feature of eukaryotic promoters (see *Results*).

### Description ability

Many biologically relevant features of regulatory systems can be easily represented with this generic model only as a matter of parametrization. In particular, it can account for multiple TFs competing for the same binding site or a TF having multiple binding sites (figure 1C1). The general formulation of the model (cf Additional file 1, §2) allows one to represent the association/dissociation of molecules either on their own or within complexes of various composition. This is an essential aspects of most ligand-receptor regulated genes (figure 1C2) where the ligand modifies the receptor's affinity with DNA and ability to recruit different cofactors. Moreover, what was so far considered a TF molecule bound or not can be generalized to represent other aspects of the state of a promoter: Alternative conformational states (DNA looping, chromatin open/closed state, nucleosome sliding ...) and the status of histone tail residues (figures 1C3-5). Furthermore, these epigenetic factors can be represented to be modified by explicit remodeling complexes and histone modifying enzymes, taking into account their essential energy-dependent and nearly irreversible nature. Also, the affinity and the enzymatic/remodeling activity of any molecule can be defined to depend on these various epigenetic factors in a combinatorial way in order to account for all the potentiality of the histone code. For instance, one can set parameters so that the acetylation of a given histone tail residue can only occur in the presence of a given histone acetyletransferase (HAT) with specific cofactors and corresponds to an highly energetic reaction. The property of epigenetic changes and promoter occupancy by TFs to occur cyclically on eukaryotic promoters [49-52,66] can be represented with various sorts of deviations from an ideal sequential recruitment (ref [71] and case (ii) in *Overview of derivations *and Additional file 1, §3.1). In the *Results *section, we show that this behavior is directly due to the energy-dependent modifications of chromatin that our model can represent. Other situations of interest can be described such as multiple copies of the same gene (figure 1C6) or two genes in the same chromatin context (figure 1C7), reproducing a situation that has demonstrated experimentally that chromosome positioning and chromatin dynamics is a key factor of noise in eukaryotic gene expression [5,6,10].

### Overview of derivations

Here we provide a short description of the theoretical derivations of this paper. For details, the reader can refer to the Additional file 1 (§3).

Several generic approaches have been proposed for deriving steady-state and/or spectral indicators of the stochastic activity of an arbitrary (sometime only linear) reaction network [23,34,69,70,74,75]. These techniques are based on a reformulation of the chemical master equation (CME) in terms of moments or on its approximation into a Langevin equation which is then solved by different methods (eg. linear noise approximation, frequency domain analysis, ...). These powerful methods could be applied to our system and may lead to similar expression of noise power as ours. However, the methods we employ here makes no approximation of the CME, thus providing exact results. It takes advantage of the fact that the CME for the whole system can be decomposed into a (finite) promoter-CME from which RNA and protein levels fluctuations are deduced using inhomogeneous Poisson birth-and-death processes. The promoter-CME is solved as a continuous-time Markov chain with standard eigenvalues-decomposition techniques [75] that have the great advantage to result in expressions of power spectra and normalized variances in terms of simple elementary components that reveal the structure of the dynamics. Note that the application of the previously mentioned generic methods on an arbitrary system as the one we consider would also require to solve a linear system or an eigenproblem.

Previous models of gene expression that incorporate a detailed description of promoter states were either solved for the steady-state mean [73] or moments [42] of expression or simulated with a Gillespie algorithm [52,67,68,76,78]. Although these simulation studies provided valuable insights into the dynamic aspects of promoter stochastic activity, the lack of analytical results do not allow for complete understanding of the dynamics and extensive exploration of parameters. Thus the dynamic aspects of promoter stochastic activity (eg. as measured by power spectra or autocorrelations) remain partially unexplored from the modeling point of view and the steady-state ones were not described in terms of distribution (a metric that reveals essential features such as number/position/size/shape of modes). Here, we derive both power spectra and distributions for an arbitrary regulatory structure.

In our model, the time-dependent vector *ϕ*(*t*) describing the probability for a promoter to be in each state evolves according to the CME(1)

where the 2^*N *^× 2^*N*^-matrix **M **represents the state-to-state transition rates (constructed from **k **in the simple version of the model and provided directly as a parameter in the general version). Diagonal elements of **M **are set so that .

The forward solution *ϕ*(*t *+ *τ*) of the CME is simply obtained from the decomposition of **M **into eigenvalues *λ*_*i *_and eigenvectors Λ_*i*_. Combined with *ρ*, it gives the autocorrelation of process *X*(*t*)(2)

from which is deduced the power spectrum *S*_*X*_(*ω*) (Eq 5).

In the general case, the eigendecomposition of **M **is obtained numerically, but we provide analytical expressions in the case of **(i) **a two-states promoter and **(ii) **a homogeneous isolated directed cycle with backward reactions, ie. a cycle of *n *states noted

In that case, the eigendecomposition(3)

shows that the spectrum of **M **consists of *n *eigenvalues regularly spaced on a circle tangent to the imaginary axis at 0 for an irreversible cycle (*k*^*b *^= 0). It flattens as an ellipse toward the real axis as backward reactions *k*^*b *^increase. Therefore the coherence of the periodic dynamics increases with the number of steps and the ratio *k*^*f*^/*k*^*b*^.

The steady-state rate of free energy change *Ė *(Eq 8) is obtained by noticing that any reaction *s*→ *s*, occurs at a rate Λ_*s*_,_0_*M*_*s*'_,_*s *_and results in a change of free energy of k_B_*T *log *M*_*s'*_,_*s*_/*M*_*s*_,_*s'*_.

Power spectra *S*_*R*_(*ω*) and *S*_*P*_(*ω*) are obtained by remarking that *R*(*t*) (resp. *P *(*t*)) is a birth-and-death process with birth rate being an inhomogeneous Poisson process with instantaneous rate *X*(*t*) (resp. *R *(*t*)). Normalized variances *σ*_X_^2^/⟨X⟩^2^, *σ*_R_^2^/⟨*R*⟩^2 ^and *σ*_*P*_^2^/⟨*P*⟩^2 ^are deduced from power spectra using the property .

Distributions of RNA levels are obtained by solving numerically the system of equations corresponding to the steady-state of the CME that considers both the promoter and the RNA level(4)

where **D**_***ρ ***_is the diagonal representation of ***ρ ***and the *s*^th ^element of *ϕ*_*r *_is the probability to have *r *RNA molecules and the promoter in state *s*.

## Results

With this model that can represent arbitrarily complex regulatory system from prokaryotes to eukaryotes, we show that the molecular interplay can cause a single gene with constant concentrations of TFs to demonstrate various forms of complex activity (eg. multiple periodicities, relaxation times) that are usually found at the network-level. We describe these activities in relation with experimental observations from the literature and highlight underlying mechanisms that might cause them and properties that they might provide.

### Spontaneous activity of an arbitrary regulatory structure

**Figure 2:**
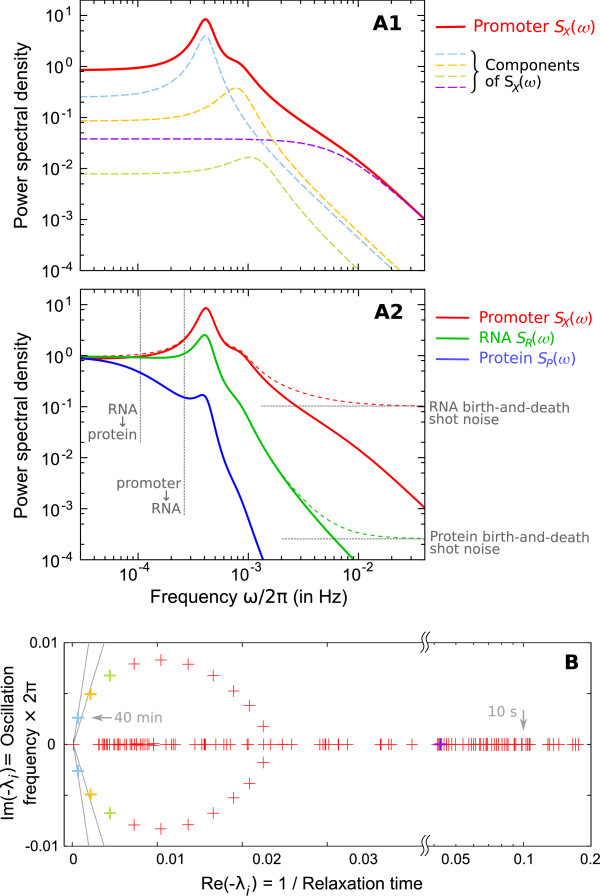
**Portrait of the regulatory structure dynamics and its transmission to RNA and protein levels**. **(A1) **The power spectrum *S*_*X*_(*ω*) of the transcriptional efficiency process *X*(*t*) (red curve) is the sum of simple components (dashed curves). **(A2) **These fluctuations of transcriptional efficiency are transmitted to RNA and protein levels undergoing at each step (cf Eq. **6**) the addition of a shot noise due to finite synthesis/degradation events (horizontal gray dashed lines: noise levels) and a low pass filtering due to time averaging (vertical gray dashed lines: cutoff frequencies). Dashed red and green curves are intermediate spectra 2*γ*⟨*R*⟩+*S*_*X*_(*ω*) and  illustrating the effect of the shot noise. **(B) **Each eigenvalue *λ*_*i *_of matrix -**M **(or pair of conjugates) corresponds to an elementary component (or mode) in (A1) and determines its characteristics (eg. frequency and thinness of the peak). For instance, the arrows correspond to a 40 min oscillation period and a 10 s relaxation time. Colored crosses identify the components displayed in (A1). Many observables on the promoter can be described by the spectrum of -**M **(cf text), making it an accurate representation of the whole regulatory structure dynamics.

We show that the power spectrum *S*_*X*_(*ω*) of transcriptional efficiency *X*(*t*) (figure 2A1) can be simply written as the sum of 2^*N *^simple elementary components (ie. elementary signals or modes)(5)

With  from the decomposition of the transition rate matrix **M **into eigenvalues *λ*_*i *_(a set of points on a plane; figure 2B) and eigenvectors Λ_*i*_.

Each eigenvalue *λ*_*i *_that lays on the real axis (ie. abscissa axis of the plane on figure 2B) corresponds to an aperiodically fluctuating signal with characteristic relaxation time -1/*λ*_*i *_determined by its position on the axis (the further from 0, the faster the fluctuations). For instance, the purple eigenvalue on figure 2B corresponds to the purple component on figure 2A1. Eigenvalues that are not on the abscissa axis come in pairs of conjugates (ie. symmetric with respect to this axis) and correspond to periodic stochastic signals which oscillating frequency |Im(*λ*_*i*_)/2*π*| is given by the ordinate of the eigenvalue (eg. light blue point on figure 2B and light blue curve on figure 2A1). The thinness of the peak in the power spectrum of the component is an important characteristic of the oscillatory behavior. It is described by the *coherence factor*

that corresponds intuitively to the number of oscillations after which two initially synchronized promoters are significantly desynchronized. This can be directly measured on experimental data such as obtained by chromatin immunoprecipitation (ChIP) [49,50,52,66], laser cross-linking [51] and fluoresence microscopy techniques [48]: the damping of the oscillations upon synchronous activation of a large number of identical promoters reflects the desynchronization among the population due to the stochastic timing in each individual cell [52]. Gray lines on figure 2B represent a coherence factors of 0.5 and 1. The most coherent eigenvalues (light blue) have a coherence of ~1.

It is important to mention that the repartition of eigenvalues on the complex plane (figure 2B) - called the *spectrum of matrix ***M **- provides a picture of the whole dynamics of the promoter. Indeed, the power spectrum of any observable (including the presence/absence of a given TF, the co-occurrence of two TFs, the contact between two chromatin proteins ..., as observed by techniques such as single-molecule FRET [81,82] for instance) can be derived similarly to *S*_*X*_(*ω*) (ie. using different values for *ρ*_*s*_) and results from the same set of eigenvalues.

Interestingly, the spontaneous activity of a single-gene with all input constant (Eq 5 and figure 2A1) is very similar to what has been indentified at the network-level [30,34] considering the interaction of several (simpler) genes. This shows that single nodes of gene regulatory networks can be much more complex than they are usually considered.

The example provided in figure 2 to illustrate the generic decomposition Eq 5 aims at reproducing the behavior of an eukaryotic promoter with realistic parameters and timescale relationships. Experimental studies of eukaryotic promoter dynamics reveal slow oscillatory patterns of occupancy and chromatin modifications with a period in the order of few tens of minutes [48-50,52,66] and a rapid turnover of TFs in the order of tens of seconds [45-48]. The transition rates **k^0 ^**were obtained using a stochastic optimization algorithm that promotes a coherent oscillatory behavior at a timescale significantly slower than the rest of the dynamics, based on criteria regarding the spectrum of **M **and with concentration of TFs set in the physiological range for eukaryotes [52,83,84], ie. 3 nM (cf table S4 in Additional file 1 for details). The resulting system demonstrates a strong cyclic activity with a period of 40 min while the fastest events occur at a timescale of less than 10 s (cf arrows on figure 2B). However, these fast events do not necessarily correspond to TF residence times and the question of how a slow global activity arises from the rapid dynamics of the molecules remains open. Most propositions from the literature suggest that at least some of these factors (eg. chromatin modifications) are slow [48,52-54].

Within the framework of our model, the particular ideal system consisting in a cycle of homogeneous directed transitions (as in [71]) produces a circle of eigenvalues in the spectrum of **M **(cf Additional file 1, §3.1.1), similarly to what results from the optimization algorithm (figure 2B). The shape and number of points of this circle indicate that the promoter progresses along a 22-steps cycle of strongly directed transitions. In comparision, [52] supposes *a priori *a cycle of 6 of such transitions and is also able to reproduce oscillations with simulations. Note that the further development regarding energy consumption we will make in this paper also argue for the physical realism of the system of figure 2.

We described the spontaneous fluctuations of promoter activity for any arbitrary promoter by a modal decomposition of the power spectrum *S*_*X*_(*ω*) (Eq 5) and illustrated it with an eukaryotic example (figure 2). Now we will focus on how these fluctuations propagate through the RNA and protein levels.

### Transmission of promoter stochasticity to RNA and protein levels

Both RNA and protein levels *R*(*t*) and *P*(*t*) follow an inhomogeneous birth-and-death process which inhomogeneous birth rates are *X*(*t*) and *R *(*t*) respectively. From this scheme, we derive the power spectra of RNA and protein levels (resp. *S*_*R*_(*ω*) and *S*_*P *_(*ω*)) from *S*_*X*_(*ω*) (cf Additional file 1, §3.3):(6)

with , ⟨*R*⟩ = ⟨*X*⟩/*γ *and ⟨*X*⟩ = *β*_0_. This expression clearly separates the different gene-intrinsic stochasticities and their physical principles from a dynamic viewpoint. In agreement with previous analyzes [34,69], each of the two steps (transcription and translation) result in (i) the addition of a *shot noise *(the classical low-copy Poisson noise) due to the finite number of random birth-and-death and determined by RNA and protein abundance and (ii) a low-pass filtering due to time averaging determined by RNA and protein life-times (figure 1A2).

Transcript and protein life-times, as reported by global analyzes on the yeast transcriptome [85] and proteome [86] for instance, are broadly distributed: from 3 min to 90 min for RNA half-lives and from less than 4 min to more than a day for protein half-lives. Interestingly, in regard with the typical period of 40~60 min of promoter cycling observed *in vivo *[48-50,52], these life-times can either dampen the promoter oscillations or let it propagate up to the protein level. Indeed, several studies showed that the RNA level can display the same cyclical patterns as observed on the promoter [48,52]. In the example of figure 1, transcript and protein life-times were chosen rather short (namely 1/*γ *= 10 min and 1/ = 25 min) and oscillation can be observed clearly in RNA fluctuations and slightly in protein fluctuations. Another global analysis in yeast [84] showed that RNA abundance ranges from less than 1 copy per cell to a hundred and that proteins are typically a thousand times more abundant. In figure 1A2, where RNA and protein abundances are ⟨*R*⟩ ≃ 30 and ⟨*P*⟩ = 1000⟨*R*⟩, the oscillations are more important than the shot noises are. Approaching 0 for RNA abundance increases the shot noise level with respect to promoter fluctuations *S*_*X*_(*ω*) and leads to the regime where the noise of RNA birth-and-death dominates.

Noticeably, the derivation from power spectrum *S*_*P*_(*ω*) of the normalized variance (a more synthetic but less descriptive indicator) reads (cf Additional file 1, §3.3):(7)

an expression similar to the well-known expression due to J. Paulsson [23] who first provided such a clear separation, but considered a set of independent two-states genes. The time-averaging coefficients and Poisson 1/⟨*P*⟩ terms directly result from the filters and shot noise terms in *S*_*P*_(*ω*).

**Figure 3:**
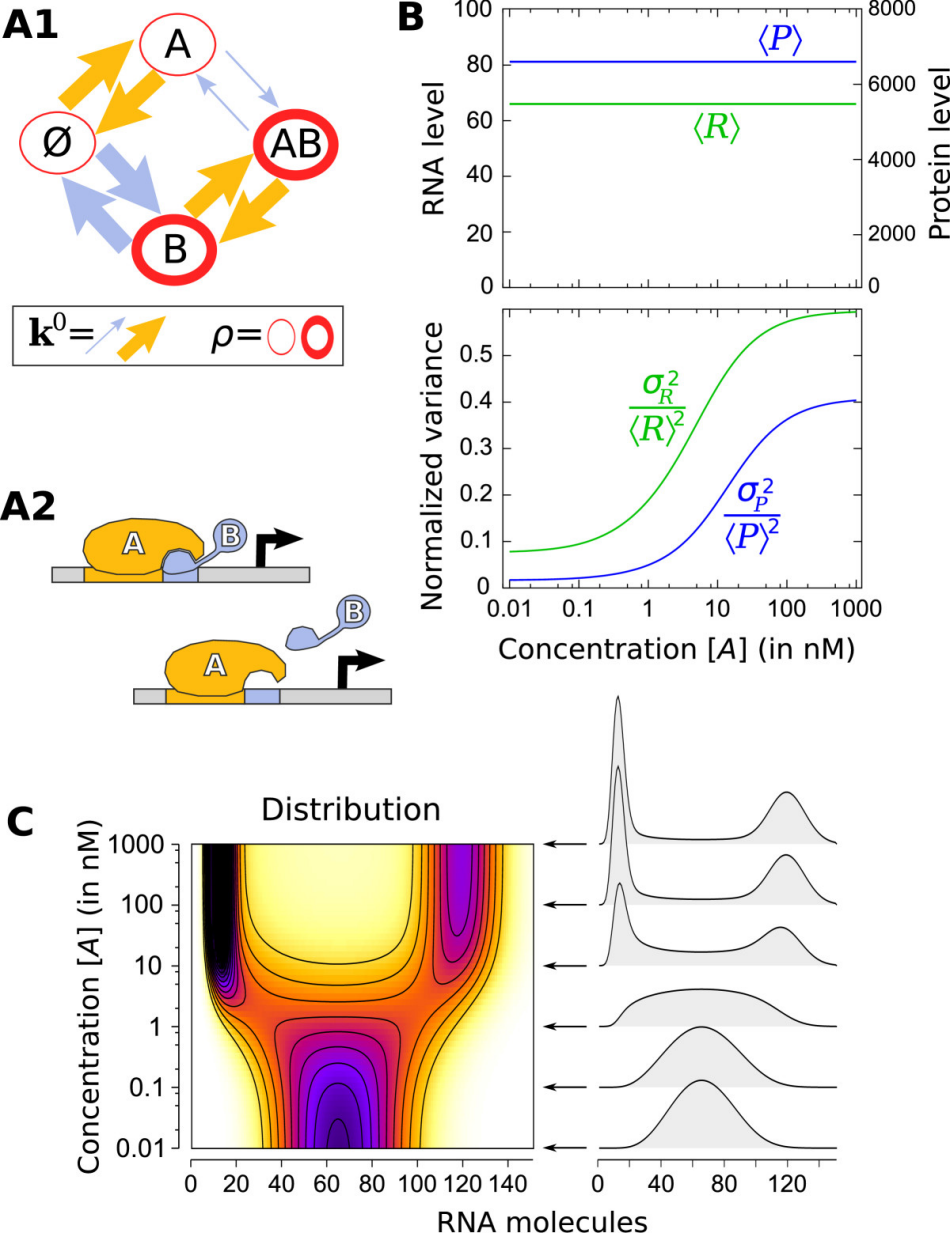
**Stochasticity induced at high concentration of a TF**. This minimalist system **(A1) **(see text or table 2 of Additional file 1 for description) that can correspond to simple molecular scenarios **(A2) **demonstrates that, contrarily to a common idea, increasing the concentration of a TF can result in a larger variability **(B)**. A more precise picture of this phenomenon is provided by observing how distribution changes with TF concentration **(C)**. Robustness of this behavior with respect to deviations from this ideal minimal model are presented in figures S1 and S2 of Additional file 1.

**Figure 4:**
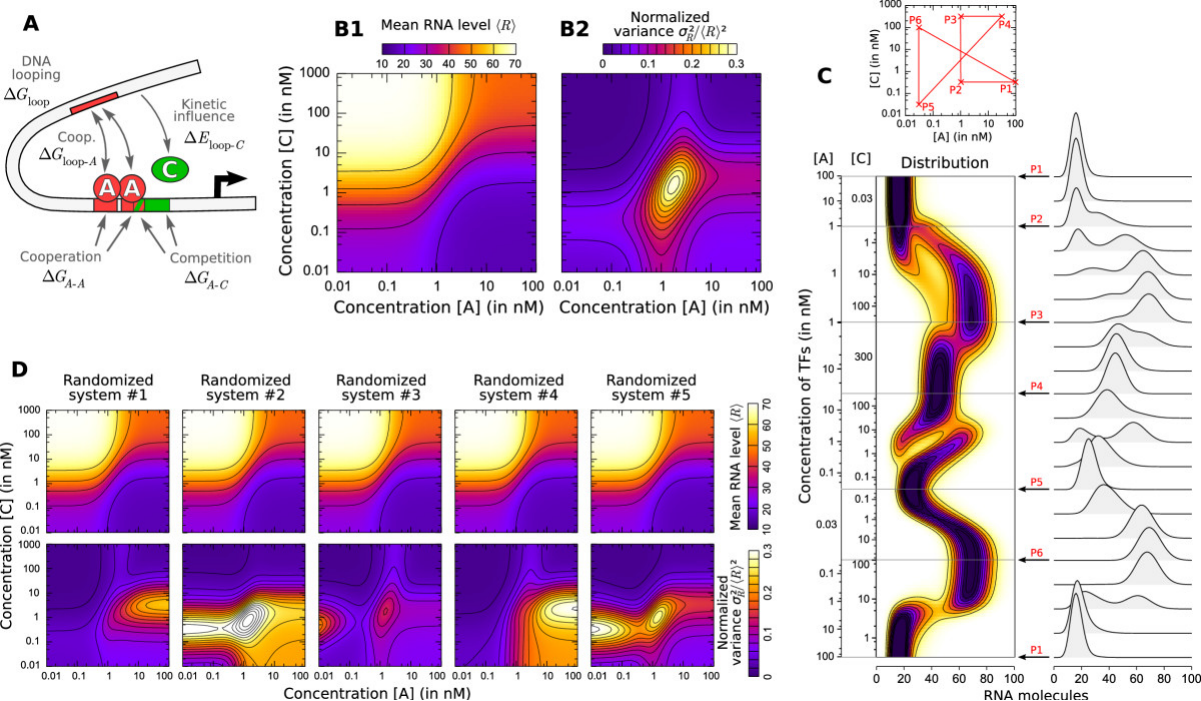
**Complexity of the steady-state**. **(A) **This prokaryotic-like example corresponds to an energy-independent promoter regulated by two TF molecules (*A *and *C*) and the looping of DNA with typical parameters from the literature. *A *binds cooperatively at its two binding sites (Δ*G*_*A*-*A *_= -2 kcal/mol) and competitively with *C *at one of its sites (Δ*G*_*A*-*C *_= 1.5 kcal/mol) [68,79]. The energetic cost of DNA looping (typically between 8 and 10 kcal/mol) is Δ*G*_loop _= 9 kcal/mol and is overcompensated by the interaction energy with two TFs of type *A *that maintain the loop (Δ*G*_loop-*A *_= -5.5 kcal/mol for each site) [67,93,94]. The closed state of DNA looping slows down the association/dissociation of *C *(Δ*E*_loop-*C *_= 2.5 kcal/mol). Bimolecular TF-DNA residence times were taken in the shorter range reported by [90] (1/ = 20 s at both sites and 1/ = 60 s) and the time for DNA to loop when both sites of *A *are occupied is very fast 1/*k*^close ^= 1 s [93]. Concentration ranges ([10^-2^; 10^3^] nM) and equilibrium constants ( = 20 nM and  = 1 nM) were set to physiological values [79,89]. Transcription is promoted by the unlooped state, the presence of *C *and slightly by the presence of *A *at one site (see table S3 of Additional file 1 for details). RNA life-time (5 min) and abundance (between 10 and 70 copies per cell) were chosen as reported by [84,91]. **(B) **Exploration of the system's behavior as a function of concentrations [*A*] and [*C*] is presented in terms of mean RNA level (B1), normalized variance (B2) and distribution **(C) **(represented along an arbitrary path of interest because of a too large dimensionality). **(D) **Changing the energies of activation **E^0 ^**by adding a normally distributed energy (s.d. = 3 kcal/mol) to both direction of each reaction while keeping state energies **G^0 ^**unchanged does not influence the mean behavior of expression but has a profound impact on its variability. This shows that mean expression can hide most of the complexity of regulation and that stochastic aspects can reveal much kinetic information.

While power spectra describe the dynamic aspects of fluctuations (eg. periodicities, relaxation times), it cannot capture several important features that reside in steady-state distributions. We provide here a numerical method for finding the full steady-state distribution of RNA molecules without any further simplification to the model (cf Additional file 1, §3.3 for details). A previous analysis proposed a method for finding the set of moments with a similar generic model for the promoter [42], but this does not reveal meaningful features such as multimodality and position/size/shape of the different modes as it will be illustrated in figures 3C and 4C.

Power spectra, normalized variance and full steady-state distribution are complementary indicators that, being derived prior to instantiation of the model, provide a comprehensive description of any arbitrarily complex regulatory system.

### Cyclical promoter occupancy and remodeling, periodic activity and energy-dependence

Our system is able to reproduce the strongly periodic activity that is observed experimentally on eukaryotic promoters [48-52,66] and referred to as cyclical recruitment or loading profile (figure 2A). This arises when the graph of promoter states contains directed cycles (ie. a closed path over which the product of kinetic constants is not the same in both directions). The very few previous works also accounting for this oscillating behavior with different modeling frameworks [52,71,72] indeed relied on a similar mechanism. We show that the absence of directed cycle in closed systems imposes that all eigenvalues are on the real axis, thus forbidding any oscillation (cf Additional file 1, §2.2 and 3.2). The signature of cycles are circles of eigenvalues in the spectrum of matrix **M **(cf *Overview of derivations *and Additional file 1, §3.1), as in figure 2B for instance. As also remarked by [52,71] but in less quantitative and/or generic terms, the essential resulting property is that the longer, the more directed and the more homogeneous the cycle, the more coherent the periodic activity.

The energetic formulation of the model (cf *Methods *and Additional file 1, §2.2) show that directed cycles are necessarily due to energy-dependent transitions in promoter state and that the more energy is consumed along the cycle, the more directed it is. The well-known property that energy-dependence is required for oscillations in metabolic networks [87] (that considers varying amount of several species in large quantities interacting through deterministic laws) can be transposed to our case (that considers the stochastic activity of a single promoter with all TFs concentration constant).

This observation has important consequences on our understanding of promoter dynamics and stochasticity. Indeed, the cyclical occupancy and remodeling profiles observed on eukaryotic promoters is highly coherent [48,50-52,66] (ie. when a large number of promoters are synchronized, it takes many oscillations before they are significantly desynchronized). In the light of our results, such a coherence requires the presence of cycles that (i) span over a very large number of states (likely several hundreds for the most coherent cases) and (ii) are highly energy-consuming. This first point is strongly supported by the profusion of possible promoter states provided by the combinatorial patterns of histone tails modifications and nucleosome positioning [60-64] and the alternate compositions of multiprotein complexes found on promoters [53,54]. The second point comes in good agreement with the fact that many ATP-dependent chromatin remodeling complexes (eg. SWI/SNF and NuRD complexes) and histone modifying enzymes (that use a cofactor as an energy donor) actively participate in the cycling behavior of active promoters [50,51]. Moreover, ATP depletion has been observed to totally suppress oscillations [51]. Hence, the oscillatory activity observed on eukaryotic promoter cannot be explained without energy consumption, a feature indeed widely present on these promoters.

The very stereotyped case consisting in a cyclic sequence of homogeneous irreversible transitions (*k*^b ^= 0 in case (ii) of *Overview of derivations *and Additional file 1, §3.1) results in a highly coherent periodic dynamics. But, considering reactions irreversible, this system theoretically consumes an infinite amount of energy. Also, in this ideal system, events of recruitment are perfectly ordered and sequential (ie. sequence is deterministic). Away from this unrealistic case, when the whole set of kinetic constants of the promoter-state graph contains directed cycles with heterogeneous transitions, alternative pathways (of various length and duration) and includes backward, incoming and outgoing transitions then the system can still demonstrate a periodic activity (ie. non-real eigenvalues). Similarly to the "preferentially random" scheme of [52], although events do not follow a predefined sequence and display a significant level of randomness, they can still tend to occur in a preferred order and result in a periodic global pattern. As a result, organizing the molecular interplay to provide the promoter with a structured dynamics (the ideas of "transcription clock" [50] and "molecular memory" [41]) has a clear energetic cost. The relevant measure reflecting this fundamental cost is the steady-state energy consumption rate:(8)

Indeed, during the optimization process used to obtain the example of eukaryotic promoter, the energy consumption rate *Ė *increases along with the coherence. For the system shown in figure 2, *Ė *equals 0.05k_B_*T*.s^-1^. The period of oscillations being 40 min and considering 20k_B_*T *for an ATP hydrolysis in physiological conditions [88], this energy consumption corresponds to the equivalent of ~6 ATP hydrolyzes per cycle. This is reasonable although real promoters are actually larger, more coherent and their energy consumption is certainly more important [50].

However, although necessary, energy-dependence is not sufficient for a periodic activity and is not the sole factor influencing the extent of this phenomenon (cf Additional file 1, §3.2). Hence, the correlation between structured promoter activity and energy-dependence is not direct, raising questions about their probably non-trivial evolutionary relation (see *Discussion*).

### Stochasticity can be induced by a high TF concentration

One possible use of this model is to identify a behavior of interest, isolate its minimal requirements and then test its applicability with realistic parameters and on larger and more biologically plausible systems. We give here an example of such use of the model.

As stochasticity is often due to the rareness of some discrete events, it is very commonly associated to low concentrations. However, we show that, in some situations, stochasticity can on the contrary be induced by a high concentration of a TF. The simplest system for this behavior to occur is described on figure 3A1. It consists of a promoter with two TFs (*A *and *B*) where the transcription rate only depends on *B*. The only mutual influence between TFs is that *B *associates and dissociates more slowly when *A *is bound to the promoter. We used realistic parameter values similar to those reported by quantitative thermodynamics [79,89] and single-molecule kinetics studies [90] of bacterial regulation for concentrations ([*A*] ∈ [10^-2^; 10^3^] nM and [*B*] = 5 nM), for bimolecular TF-DNA residence times (1/ = 30 s and 1/ = 60 s) and equilibrium constants ( = 0.5 nM and  = 5 nM) and for modification of activation energy upon interaction (Δ*E *= 2.5 kcal.mol^-1^). RNA and protein life-times are 5 and 20 min [91]. All parameters are summarized in table S2 of Additional file 1.

This simple system demonstrates not only that a TF can regulate the variance of RNA and protein levels without influencing their mean (as previously identified with various mechanisms [28,42]), but more interestingly, that normalized variance can increase with the concentration of a TF (figure 3B). Indeed, here it is at high concentrations of *A *that events influencing transcription (ie. *B *associations and dissociations) become rare and, as it has been known for a long time [2,3,14,23-27,30,32,34-37,40], slow promoter dynamics result in strong variability. RNA distribution goes from unimodal to bimodal as [*A*] increases (figure 3C). Thus, the oversimplistic assumption that increasing the concentration of a TF necessarily reduces the stochasticity is not always valid.

Moreover, this property appears to be quite robust when exploring all sorts of deviations from this ideal case (eg. considering a different concentration for [*B*], an influence of *B *on *A*, a dependency of the transcription rate on *A *..., figure S1 in Additional file 1), and even for a more complex regulatory system with a larger number of TFs and randomly drawn parameters (figure S2 in Additional file 1).

Simple molecular scenarios can be imagined that would give rise to this behavior. For instance, the shape of a TF (type *A*) can be so that, when bound, it prevents other TFs (type *B*) from association/dissociating from the promoter (figure 3A2). Another example would be to consider *B *the chromatin state and *A *a TF that binds to the same site as chromatin remodeling complexes. Then, designing molecular constructions to verify this hypothesis experimentally appears to be promising.

### Potentiality of the molecular interplay

**Figure 5:**
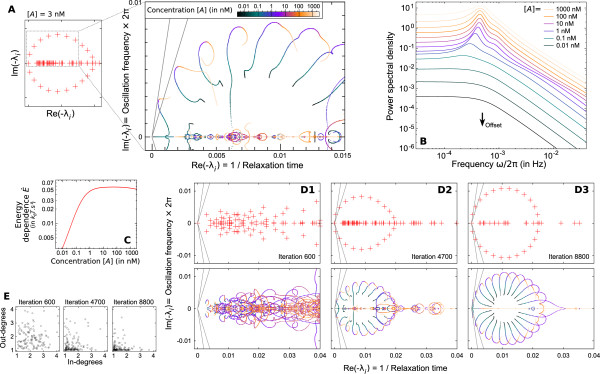
**Complexity of the dynamics**. **(A) **The concentration-dependence of promoter activity for the same system as in figure 2 is described by the trajectories of eigenvalues -*λ*_*i *_on the complex plane, showing how the different periodic and aperiodic components of the dynamics are modified (eg. frequency, coherence). **(B) **They have a direct impact on the power spectrum of transcriptional activity *S*_*X*_(*ω*). **(C) **Energy consumption rate *Ė *also varies significantly with [*A*]. **(D) **As a given system is optimized for a coherent periodic activity (D1-3 are different stages of a given optimization), the trajectories of its eigenvalues tend to stereotype. In between a highly disordered system **(D1) **and a system close to an homogeneous irreversible cycle **(D3)**, the promoter can demonstrate both a significant coherence and a complex concentration-dependence **(D2)**. The comparison of these three systems in terms of in- and out-degrees of promoter states illustrate the structuring of the transition graph toward a unique path (in- and out-degrees of a given state are taken as the sum over the maximum of its in- and out-transitions respectively).

Using this generic model and the prediction of the different indicators we provided allows us to explore in detail the activity of regulatory systems on large ranges of concentration. To illustrate the potentiality of steady-state and dynamic properties, we consider here two examples (figures 4 and 5) dedicated to represent respectively a prokaryotic promoter - with typical features as in [56,67,80] - and a eukaryotic promoter (the same as in figure 2) - reproducing the periodic behavior as observed in [48-52,66]. Although it is the expected behavior that complex activities can arise from large systems with many parameters, we show that such behavior can occur with physically realistic parameters and be due to relevant biological mechanisms. We further argue in *Discussion *that several typical features of eukaryotic promoter are precisely those that give rise to a complex dynamics. We also show how this complexity is hidden by common measures and/or modeling frameworks and identify what kind of features can modulate or constrain it.

#### Steady-state aspects

Representing the behavior of a gene as a function of TF concentrations by a combination of Hill functions is very common and often matches experiments [92]. However, we show that considerable complexity can be hidden beyond this apparently simple mean activity.

Figure 4A describes a prokaryotic promoter regulated by a TF *A*, binding cooperatively at two binding sites and competitively with a TF *C*. It also considers the looping of DNA, influencing and influenced by both TFs. All parameters were set to realistic values according to the experimental literature of prokaryotic regulation [67,79,84,89-91,93,94]. They are described in detail in the caption of figure 4 and summarized in table S3 of Additional file 1. In this system, while the mean RNA level as a function of the two TF concentrations reproduce the classical Hill-like four plateaus observed in *in vivo *experiments [92] (figure 4B1), the normalized variance (figure 4B1) and distribution (figure 4C) reveal a significantly more complex profile carrying much information. Thermodynamic models that only describe the promoter by a free energy **G^0 ^**for each state [79,80] are only able to predict the mean of gene expression. Interestingly, in our system, randomizing the activation energies **E^0 ^**while keeping the state energies **G^0 ^**unchanged does not affect the mean expression at all but has a dramatic impact on the stochastic aspects (figure 4D). This shows that the stochasticity of gene expression, even measured with steady-state metrics, is controlled by - and hence can reveal - the dynamics of the promoter. Such an observation not only has strong experimental implications, but also shows that the mean level is not the sole aspect of gene expression that can be regulated and that an elaborate control of the stochastic aspects can also be achieved (cf *Discussion*).

#### Dynamic aspects

The dynamic aspects of promoter activity and in particular the periodic cyclical promoter occupancy and remodeling patters can demonstrate a complex concentration-dependent behavior. We showed earlier that the position of the eigenvalues *λ*_*i *_(figure 2B) reflects the dynamics of the whole regulatory structure. Each *λ*_*i *_determines the correlation time of aperiodic fluctuations or the frequency and coherence of periodic fluctuations as elementary components of the global dynamics. Since the matrix **M **depends on TF concentrations, the position of its eigenvalues change with the concentration of a TF. As illustrated on figure 5A with the same system as in figure 2, these eigenvalues can demonstrate non-trivial trajectories. They describe how the temporal characteristics of the periodic loading patterns of molecules change with concentration of TFs and have a direct impact on the resulting transcriptional activity *S*_*X*_(*ω*) (figures 5B). Indeed, the basic fact that the concentration of a given TF impacts its association rate not only affects the duration of specific states in the cycle (hence influencing both frequency and coherence), but it also changes the relative probability between the transitions leaving these states. This can have a large variety of effects: influencing the amount of backward reactions in cycles, the probability of outgoing transitions (that can temporarily lead to a local dead-end or to abortion of the cycle), the balance between alternative pathways within a given cycle, and even the commitment of the system between multiple dynamics. Each one of these phenomena has its own effect on the dynamics. In simple ideal systems, these effects could be predicted, but in non-stereotyped system, they can all occur together at different points and with various intensities, resulting in a non-trivial and highly non-linear activity and providing the dynamics with a strong plasticity. Indeed, eigenvalues move along trajectories that can include sharp bends and bifurcations (inducing threshold effects) and with very variable velocity (implying different sensitivity in different concentration ranges). Also, we see that, in this context, energy consumption rate *Ė *is related but non-directly correlated to the periodic activity of the promoter and that other factors may play a role in their relation (figure 5C).

Figures 5D and 5E present three different promoters obtained at different stages of the optimization process used to construct the system of figure 2. The presented systems range from a very unstructured promoter where states have many in- and out-transitions (figure 5E1) to a stereotyped system close to the homogeneous cycle as indicated by the clear circle on figure 5D3 and the fact that most states have a unique outgoing transition (figure 5E). The decreasing complexity observed along the optimization process suggest a tradeoff between the coherence of the periodic activity and the flexibility of its regulation. Interestingly, it is at an intermediate level of structuring (figure 5D2), where the system still retains a certain level of disorganization, that a promoter can achieve a significantly coherent dynamics that nonetheless demonstrates a complex concentration-dependent behavior. We argue in *Discussion *that different features observed on real promoters correspond to this intermediate regime.

## Discussion

### The promoter as a central piece in the control of stochasticity

Stochasticity in gene expression can be advantageous in some situations and harmful in others [15-21]. Therefore, it appears that it should take place at specific times (eg. in response to external factors), on specific genes and with a certain form. We showed how the dynamics of the regulatory structure can induce a large variety of stochastic activities on which a tight control can be exerted. Other mechanisms identified at other stages of the expression process have been shown to influence stochasticity [23,24,27-29,31-34,38,39,41], but none of them provide such a diversity and such a control. Regulation has long been considered as a way to control the mean expression, but it results to be a very powerful and flexible tool for the cell to take advantage of stochasticity by modulating it depending on the context.

Moreover, these capacities require no particular unusual molecular machinery and can be obtained in realistic parameter ranges. They only rely on common features of living systems such as protein-DNA and protein-protein interactions, conformational changes, energy-dependent activity of molecules and, more specific to eukaryotes, modifications of chromatin structure and epigenetic state of histones. Hence, controlling stochasticity is not only possible, but it also appears to be easy to achieve.

### Limitations and future work

Pedraza *et al*. [41] described the effect on *σ*_*P*_^2^/⟨*P*⟩^2 ^of considering molecular memories (ie. non-exponential waiting times) on a two-state on/off promoter (represented phenomenologically by bursts of arbitrarily distributed size and waiting time intervals) and on RNA and protein lifetimes. Our work can be viewed as a deep focus on the causes and consequences of the molecular memory that takes place at the promoter. But the fact that indeed RNA and proteins degradations are not first order (the ubiquitin-proteasome system induces non-exponential lifetimes), but also that synthesis are not instantaneous (elongation, splicing, nuclear export ... induce distributed delays) influences the shape of filters in Eq 6 and hence the normalized variance. However, these phenomena only constitute deviations (although potentially strong) from the global tendency that our simple description of these steps can capture.

Protein-protein interactions can occur away from the promoter and can result in the binding of complexes to the promoter (eg. ø → *AB*) as well as TFs unbinding as complexes (eg. *AB *→ ø). This is taken into account in the general version of the model (cf Additional file 1, §2). In that case, concentrations of complexes are not null and simply imply a larger set of kinetic constants than **k**^0^. Still, once the matrix **M **is constructed, all the mathematical derivations are strictly identical. Off-promoter TFs associations/dissociations (eg. modeled by a simple reaction network) determine the concentration of free TFs (ie. [*A*], [*B*], [*C*] ...) and of complexes (ie. [*AB*], [*BC*], [*ABC*] ...) so that varying the amount of one TF potentially results in variation of concentrations of all TFs and complexes. In particular, if on-promoter and off-promoter interactions are different, this results in the coupling of two different dynamics, leading to additional complexity in eigenvalues trajectories and providing even more flexibility for regulating the dynamic activity of promoters.

This model of gene expression focuses on intrinsic stochasticity of non-autoregulated genes. Going further by considering extrinsic stochasticity and autoregulation could be achieved with various techniques [23,34,69,70,74,75]. In the light of the highly non-linear spontaneous behavior we showed, revisiting with the present model properties of signal transmission and stochastic resonance that have been identified with simple models of promoter [22,28-30,32,38,39,43] can already be expected to reveal new properties of gene regulatory structures.

### Back to experimentation: what can be expected?

The *in vivo *activity of promoters can be explored experimentally as a function of TFs concentrations [73,92]. Typically, Setty *et al*. [92] measured the mean RNA levels of the *lac *operon of *E. coli *as a function of cAMP and IPTG concentrations and interpreted their data in terms of Hill functions. We showed that the steady-state stochastic aspects of expression (that can be obtained by flow cytometry) carry much more information (figure 4). The normalized variance demonstrates a more characteristic profile than the mean level and the distribution itself carries several singularities (varying number/position/size/shape of modes), constituting a real signature of the promoter activity. Indeed, different systems can be obtained showing a large variety of stochastic profiles for a strictly identical mean activity (figure 4D). However, inferring parameters from such measurements appears conceivable only for rather simple prokaryotic promoters for which some information is already partially known. Nevertheless, it can reveal the kinetic information that is known to be difficult to obtain [68]. Focusing on mean equilibrium activity, thermodynamic approaches can only infer ratios of pairs of kinetic constants (ie. equilibrium constants) and therefore only state energies **G**^0^. On the contrary, our approach can provide the kinetic constants themselves and reveal the energies of the activation barriers **E**^0^.

The greater complexity of eukaryotic promoters certainly moderates the success of the previous approach. Nevertheless their singular dynamics promotes the use of time-dependent measures - as obtained by photobleaching [46-48,55], chromatin immunoprecipitation [49,50,52,66] or laser-crosslinking techniques [51] - and ask for the investigation of their concentration-dependent behavior. This would reflect, although partially, trajectories of eigenvalues *λ*_*i *_(figure 5A1). For instance, frequency and damping of cyclical occupancy patterns have been observed to change strongly with inducer concentration (R. Métivier, personal communication). Information on the matrix spectrum can also be obtained by timelapse microscopy measurement to follow across time the amount of proteins or transcripts. Giving access to spectral measurements such as *S*_*R*_(*ω*) or *S*_*P*_(*ω*), this technique was shown to give mechanistic insight into the underlying system [7,8,12,95].

This generic model can thus constitute a unified theoretical framework for all these very different techniques, making them complementary views of the same system.

### High mobility, functional redundancy and alternate promoter states

It appears from our study that a very constrained system that consists in a unique cyclic sequence of events (as [71] and case (ii) in *Overview of derivations*) results in a simple and stereotyped concentration control ability (eg. figure 5D3). On the other hand, a very random transition graph can demonstrate complex trajectories of eigenvalues but lacks of a coherent periodic activity (eg. figure 5D1). It is when the system has long directed cycles (requiring the presence of energy-dependent transitions) but retains a certain level of disorganization (ie. cycles are not restricted to a unique homogeneous sequence of events but can contain heterogeneities and alternative pathways of variable length and kinetic characteristics) that a complex control of a sensibly coherent periodic activity can occur (eg. figure 5D2). It is worth noting that similar characteristics of molecular interactions have also been proposed to account for a different phenomenon: the generation of a slow population dynamics from rapid molecular kinetics [52]. Interestingly, such properties correspond to what is actually observed experimentally [48,53,54]: Within a slow and coherent periodic dynamics, multiprotein complexes are very unstable due to transient and dynamic interactions of most proteins and can therefore be in a variety of alternate composition. The various proteins species, which recruitment varies along the slow cycle, can have different mobility, therefore inducing kinetic heterogeneities in the slow cycling dynamics. Also, different subcomplexes have been shown to be mutually exclusive but functionally redundant and to form alternatively on the promoter at the same point in the slow cycle, corresponding to stochastic temporary commitments between distinct pathways [50,53,54]. This depiction of the dynamics of eukaryotic regulatory structures is likely to result in a complex and flexible concentration dependence. It can even be speculated to have evolved to provide this powerful control mechanism.

### The evolutionary cost of the "transcription clock"

Cyclical patterns of promoter remodeling and occupancy by TFs are often associated to the idea of "transcription clock" [50] for its tendency to provide the dynamics with a certain timing and to synchronize cells in response to external factors. However, as we have shown, imposing periodicities in the dynamics of the regulatory structure requires energy and is limited by the number of possible promoter states. The clear cyclical recruitment of TFs observed *in vivo *on eukaryotic promoters and its strong coherence indicate a large number of steps and a strong energy-dependence.

Moreover, any deviation from the stereotyped cycle results in a diminution of the coherence of oscillations and makes it suboptimal with respect to energy consumption. This raises an indirect additional cause of energy cost: Achieving a complex concentration-dependent activity (requiring many of such deviations) while keeping a coherent periodic dynamics could represent another evolutionary pressure toward stronger energy consumption and larger regulatory structures.

## Conclusion

In the light of the various capabilities of gene promoters we have demonstrated in this study, it seems that the importance of the single-gene level has to be reconsidered. Indeed, systems biology has set gene networks to the front of the stage, expecting complexity to arise from the interaction of many genes, often considered simple and deterministic. It appears now that single nodes of these networks should be given more attention since their spontaneous stochastic dynamics can be a considerable source of complexity.

## Authors' contributions

AC, OG and GB designed the study. AC performed the theoretical derivations of the model and conceived/analysed the different systems presented as examples. AC, OG and GB interpreted the results and wrote the paper. All authors read and approved the final manuscript.

## Supplementary Material

Additional file 1 **Supplementary information**. This document contains two supplementary figures, an extensive description of the model in different versions and formulations, details of theoretical derivations and all the parameter values of each system presented as example in the main article. Click here for file
