# Supplementary material for: On the spontaneous stochastic dynamics of a single gene: complexity of the molecular interplay at the promoter
Source: BMC Syst Biol. 2010 Jan 8;4:2. doi: 10.1186/1752-0509-4-2 (PMC2832887; doi:10.1186/1752-0509-4-2)
Supplement: Additional file 1 — Supplementary information. This document contains two supplementary figures, an extensive description of the model in different versions and formulations, details of theoretical derivations and all the parameter values of each system presented as example in the main article. [file 1752-0509-4-2-S1.PDF]

On the spontaneous stochastic dynamics of a single gene: complexity of the molecular interplay at the promoter

A. Coulon, O. Gandrillon and G. Beslon

Supplementary Information

Contents

1 Supplementary figures **2**
Supplementary figure S1 . . . 2
Supplementary figure S2 . . . 2

2 Definition of the model **3**
2.1 Kinetic definition . . . 3
2.2 Energetic reformulation and generalization of thermodynamic approaches . . . 4

3 Theoretical derivations **5**
3.1 Promoter dynamics . . . 5
3.2 Implications of energy consumption . . . 7
3.3 Transmission to RNA and protein levels . . . 7

4 Parameters **9**
Table S1: Parameters for Figure 1 of the main article . . . 9
Table S2: Parameters for Figure 3 of the main article . . . 10
Table S3: Parameters for Figure 4 of the main article . . . 10
Table S4: Parameters for Figures 2 and 5 of the main article . . . 10
4.1 Optimization algorithm for the eukaryotic promoter example . . . 11

5 References **11**

Notes:

- If not mentionned otherwise, sums are performed over sets that are implicitly $f \in \mathcal{F}$ the N transcription factors (TFs), $s \in 2^{\mathcal{F}}$ the 2^N promoter states (power set of $\mathcal{F}$) and i from 0 to $2^N - 1$, the 2^N eigenvalues of $\mathbf{M}$
- We simplify the notation of promoter states by noting for instance $\{A, C\}$ as AC and $\{f\}$ as f
- The symbol $\ominus$ denotes the symetric difference between sets: $s \ominus s' = s \cup s' - s \cap s'$
- Units: RNA and protein levels are given as number of molecules (integer values) and TFs quantities as concentrations.

1 Supplementary figures

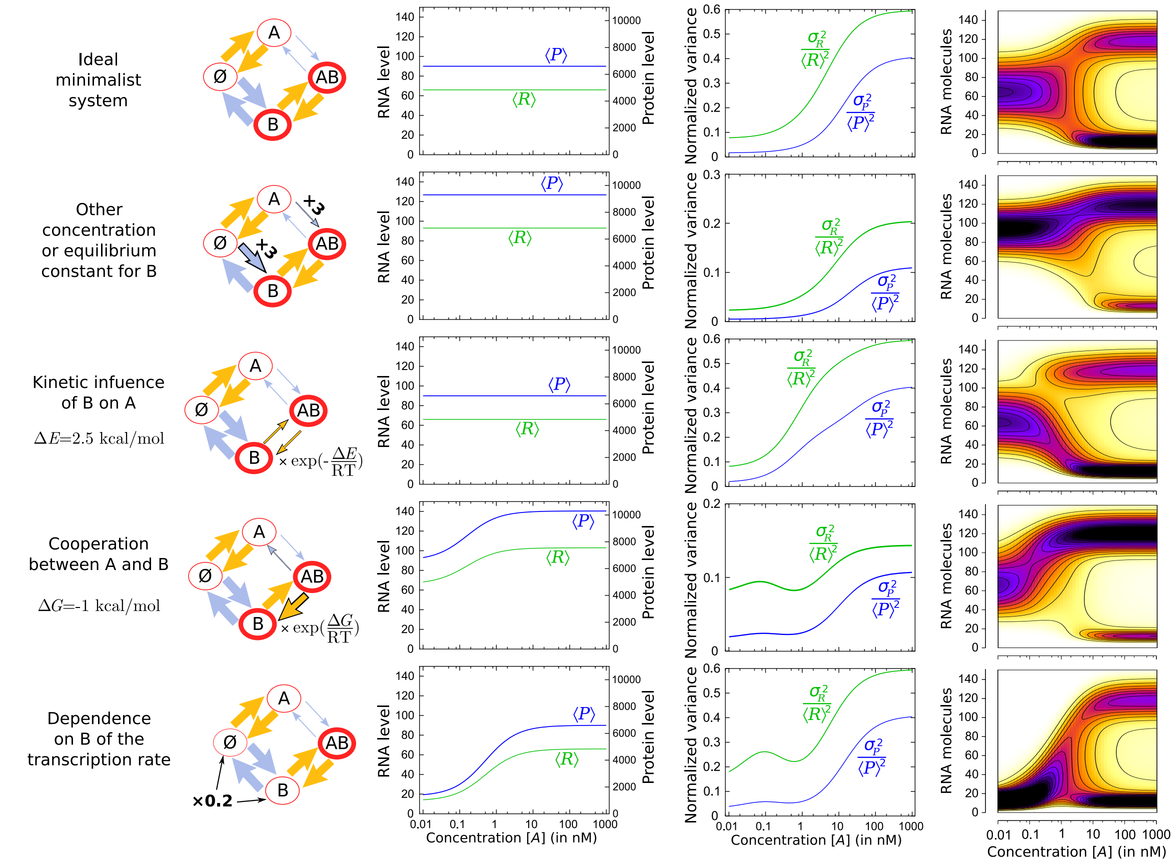

Figure S1: **Relaxation of independencies and symmetries.** The robustness of the identified property can be tested with respect to deviations from the ideal minimal system by changing concentration $[B]$, introducing a dependence on B on transitions of A, a cooperation between A and B and or a dependence on A of transcription rate. As in the main article, the behavior of the system is presented in terms of mean, normalized variance and distribution.

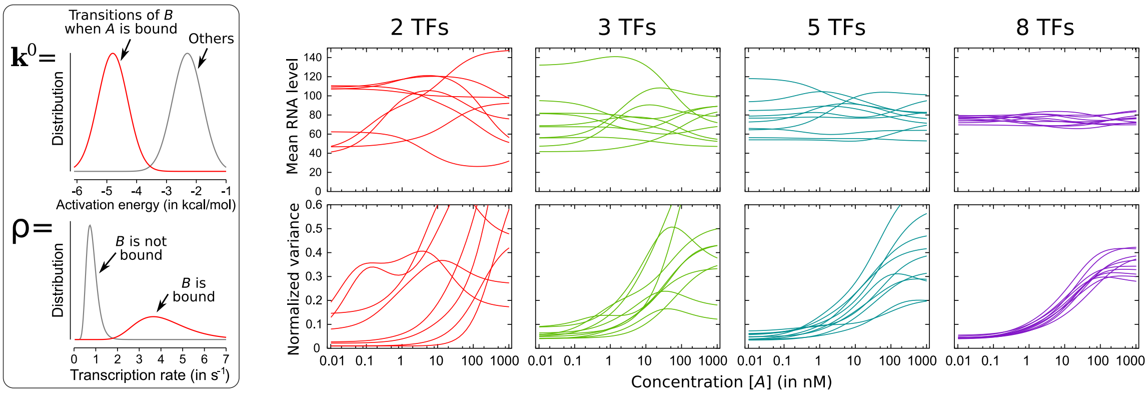

Figure S2: **Distributed parameters and larger systems**. Association/dissocation rates $\mathbf{k}^0$ and transcription rates $\boldsymbol{\rho}$ are drawn from log-normal probability distributions (*left panel*). We inspect mean $\langle R\rangle$ and normalized variance ${\sigma_R}^2/\langle R\rangle^2$ for systems with 2, 3, 5 and 8 TFs (10 random draws each). It shows that stochasticity increases with $[A]$ as long as B associations/dissociations (be they distributed) are sensibly faster when A is unbound and transcription rates sensibly depend on B.

2 Definition of the model

2.1 Kinetic definition

2.1.1 General case

The model describes the stochastic association and dissociation of an arbitrary number N of transcription factors (TFs) on the promoter of a gene. Note that, as argued in the main article, this notion of TF can be generalized to represent also various epigenetic factors. The set of transcription factors is denoted $\mathcal{F}$ and the set of possible states of the promoter is denoted $2^{\mathcal{F}}$ (the powerset of $\mathcal{F}$). For instance, for a promoter having $N = 3$ TFs $\mathcal{F} = \{A, B, C\}$, promoter states are $2^{\mathcal{F}} = \{\varnothing, A, B, C, AB, AC, BC, ABC\}$.

The model corresponds to the following set of chemical reactions:

$$\begin{cases} \mathcal{G}_s & \xrightarrow{M_{s',s}} & \mathcal{G}_{s'} & , s \neq s' \\ \mathcal{G}_s & \xrightarrow{\rho_s} & \mathcal{G}_s + \mathcal{R} & , \forall s \\ \mathcal{R} & \xrightarrow{\gamma} & \varnothing & \\ \mathcal{R} & \xrightarrow{\tilde{\rho}} & \mathcal{R} + \mathcal{P} & \\ \mathcal{P} & \xrightarrow{\tilde{\gamma}} & \varnothing & \end{cases} \tag{1}$$

where $\mathcal{R}$ and $\mathcal{P}$ are RNA and protein molecules and $\mathcal{G}_s$ is the gene of interest with its promoter in state s (eg. $\mathcal{G}_{AC}$ represents the promoter with only TFs A and C bound to their target site). The lines of this chemical system correspond respectively to a change in promoter state (due to an association or a dissociation of a TF or a complex of TFs), transcription (with a rate ρ_s that depends on the currents state s of the promoter), RNA degradation, translation and protein degradation.

We note $X(t)$ the time-dependent transcription rate (if $s(t)$ is the state of the promoter at time t, then $X(t)$ is simply the value of $\rho_{s(t)}$). We also note $R(t)$ (resp. $P(t)$) the RNA (resp. protein) level; ie. the number of $\mathcal{R}$ (resp. $\mathcal{P}$) molecules.

Each state-to-state transition rate $M_{s',s}$ (being an association rate if $s' \supset s$ and a dissociation rate if $s' \subset s$) corresponds to a kinetic constant (noted $M^0_{s',s}$) multiplied, only in the case of associations, by the concentration of the TF (ie. $[A]$, $[B]$ and $[C]$) or the complex (ie. $[AB]$, $[AC]$, $[BC]$ or $[ABC]$) that associates (namely $s' \ominus s$). For instance:

- $M_{ABC,AB} = [C]M^0_{ABC,AB}$ is the association rate of TF C when only A and B are on the promoter.
- $M_{AB,ABC} = M^0_{AB,ABC}$ is the dissociation rate of TF C when the other TFs on the promoter are only A and B.
- $M_{ABC,A} = [BC]M^0_{ABC,A}$ is the association rate of complex BC when only TF A is on the promoter.
- $M_{A,ABC} = M^0_{A,ABC}$ is the dissociation rate of complex BC when A is the only other TFs on the promoter.

Because an association and a dissociation cannot occur at the same time, all $M_{s',s}$ where neither $s' \supset s$ nor $s' \subset s$ but $s' \neq s$ are necessarily null (eg. $M_{AB,BC} = 0$).

This defines the $2^N \times 2^N$ matrix $\mathbf{M}$ that contains all the transition rates of promoter state, incorporating all the kinetic constants (defined in matrix $\mathbf{M}^0$) and all the concentrations of free TFs (ie. that are not in complexes) and complexes. For calculation reasons (cf §3.1), diagonal elements of $\mathbf{M}$ and $\mathbf{M}^0$ (where $s' = s$) are set so that the sum over each colum is null.

For determining concentrations of free TFs and complexes, one can define as parameters of the system the total concentrations of TFs (either free or within complexes), noted $[f^*]$ for a TF $f \in \mathcal{F}$ (eg. $[B^*] = [B] + [AB] + [BC] + [ABC]$), and a set $\mathbf{\Theta}$ of chemical reaction describing the interactions of TFs away from promoter. For instance, $\mathbf{\Theta} = \{A + B \overset{K=3}{\rightleftarrows} AB, A + C \overset{K=.1}{\rightleftarrows} AC\}$. As all TFs are considered in large quantities, concentrations of free TFs and complexes can be obtained by considering the steady-state of the chemical system $\mathbf{\Theta}$.

2.1.2 Without complexes (un)binding and off-promoter TFs interactions

As in the description provided in the main article and as in [1], it possible to simplify the model by considering only cases where no interactions occurs between TFs away from promoter (in other words

$\mathbf{\Theta} = \varnothing$) and where TFs cannot associate/dissociate with the promoter as complexes (meaning that $M^0_{s',s} = 0$ for $|s' \ominus s| > 1$).

In that case, TFs always being free, $[f^*] = [f], \forall f \in \mathcal{F}$ and therefore the N-vector $[f]_{f\in\mathcal{F}}$ is directly a parameter of the system. Also, the definition of the $2^N \times 2^N$-matrix $\mathbf{M}^0$ where some values are free parameters and others are imposed can be simplified to a smaller $N \times 2^N$-matrix $\mathbf{k}^0$ where all parameters are free. $k^0_{f,s}$ represents the association or dissociation constant of TF f when the promoter is in state s. Similarly, a $N \times 2^N$-matrix $\mathbf{k}$ is obtained by multiplying association rates by TFs concentrations. Then, matrix $\mathbf{M}$ is simply a reorganization of $\mathbf{k}$.

All the theoretical derivations of §3 are based on matrix $\mathbf{M}$ and are applicable for both cases.

2.2 Energetic reformulation and generalization of thermodynamic approaches

Regulation is classically approached with thermodynamic models [2, 3, 4]. These only focus on the equilibrium and mean behavior of necessarily closed systems and cannot provide a description of any kinetic aspects nor represent open systems (ie. that consume energy; as it is the case of most eukaryotic promoters). We show how our kinetic approach constitutes a generalization of the classical thermodynamic approaches to regulation. Although it requires more parameters, it makes it possible to study the stochastic and dynamic aspects of promoters that can include energy-dependent transitions (eg. ATP hydrolysis).

The master equation formalism, that a kinetic formulation makes possible to apply (cf §3), has been extensively studied [5, 6] and in particular its energetic signification and the relations between energy consumption, cycles and oscillations. Here, we apply these notions in the context of gene regulation.

We consider the general formulation of the model provided in §2.1.1 that includes the simpler version described in the main article.

2.2.1 Classical thermodynamic formulation

Thermodynamic approaches consist in defining a Gibbs free energy differences ΔG for the association of each TF to DNA and for interactions between TFs (for any pair, triplet, ... of TFs) [3]. This is equivalent to define a free energy for all the possible states and corresponds, for our system, to the 2^N-vector $\mathbf{G}$.

However, defining $\mathbf{G}$ only describes the equilibrium constants for all transitions, that is the ratio between the two kinetic constants (forward and backward) of the considered reaction. For a transition from s to s', the equivalence is given by

$$G_{s'} - G_s = -\mathrm{k_B} T \log \frac{M_{s',s}}{M_{s,s'}} \qquad (2)$$

$\mathrm{k_B}$ is the Boltzmann constant[1] and T the temperature. Moreover, by construction, this definition restrains the matrix $\mathbf{M}$ to be free of directed cycles. Indeed, for any cycle consisting of a sequence of n states noted $s_0, s_1, \ldots, s_{n-2}, s_{n-1}, s_n$ (with $s_0 = s_n$), considering implicitly a closed system, the free energy difference along the cycle $\sum_{p=0}^{n-1} G_{s_{p+1}} - G_{s_p}$ is necessarily null. Thus, $\sum_{p=0}^{n-1} -\mathrm{k_B} T \log(M_{s_{p+1},s_p}/M_{s_p,s_{p+1}}) = 0$, which rewrites

$$\prod_{p=0}^{n-1} M_{s_{p+1},s_p} = \prod_{p=0}^{n-1} M_{s_p,s_{p+1}} \qquad (3)$$

So the product of kinetic constants along any cycle is always the same in both directions.

2.2.2 Introducing kinetics

The knowledge of kinetic constants themselves (not their pairwise ratios) requires the energy of the activation barrier between any two states. We note $\hat{E}_{s',s}$ the energy of the activation barrier between

[1] Thermodynamic approaches often consider molar quantities and hence use the ideal gas constant R instead of the Boltzmann constant. As we consider a single promoter, we prefer to use $\mathrm{k_B}$.

states s and s', stored in a $2^N \times 2^N$-matrix $\hat{\mathbf{E}}$ (where only values for which $s' \supset s$ or $s' \subset s$ are defined), considered symmetric for now $\hat{E}_{s',s} = \hat{E}_{s,s'}$. Then, kinetic constants follow from the Arrhenius equation[2]

$$\hat{E}_{s',s} - G_s = -\mathrm{k_B} T \log M_{s',s} \tag{4}$$

And indeed, because $\hat{\mathbf{E}}$ is symmetric, equation (4) implies equation (2).

2.2.3 Representing open systems

During the reaction $s \rightarrow s'$, for passing the activation barrier, the system receives a certain amount of energy from thermal fluctuations of its surrounding (ie. $\hat{E}_{s',s} - G_s$). Then, relaxing from the activation barrier to the state s', it immediately releaves another amount of energy (ie. $\hat{E}_{s,s'} - G_{s'}$). In the case of closed systems ($\hat{E}_{s',s} = \hat{E}_{s,s'}$), the resulting energy difference is simply the free energy difference between the two states ($\Delta G = G_{s'} - G_s$). Then, when the system returns in state s (either directly or through a different pathway), the system will have received and released exactly the same amount of energy.

Considering now the system to be open and to receives chemical energy (eg. hydrolysis of an ATP molecule) during reaction $s \rightarrow s'$ simply implies that $\hat{E}_{s',s} < \hat{E}_{s,s'}$ (the difference $\hat{E}_{s,s'} - \hat{E}_{s',s}$ being the amount of chemical energy received). Then, when returning in state s (either directly or not) the system will have released more thermal energy than it received, but the surrounding will have lost chemical energy (eg. due to ATP degradation).

Then, as matrix $\hat{\mathbf{E}}$ is not symmetric anymore, we have, for any cycle

$$\sum_{p=0}^{n-1} \hat{E}_{s_{p+1},s_p} - \hat{E}_{s_p,s_{p+1}} = -\mathrm{k_B} T \log \prod_{p=0}^{n-1} \frac{M_{s_{p+1},s_p}}{M_{s_p,s_{p+1}}} \tag{5}$$

Therefore, a cycle is directed if and only if it includes reactions that consume chemical energy (and releases of chemical energy, if any, do not counterbalance consumptions).

This property of an energy-independent system to be free of directed cycle is commonly known as the detailed balance property [5, 6] (also known as reversibility of the Markov chain, micro-reversibility or abscence of circulation) and refers to the fact that, in that case, each reaction occurs equally in both directions $M_{s',s}\Lambda_{s,0} = M_{s,s'}\Lambda_{s',0}$ ($\Lambda_{s,0}, \forall s \in 2^{\mathcal{F}}$ is the steady-state of the system).

3 Theoretical derivations

All derivations in this section stand whatever the definition of matrix $\mathbf{M}$ (cf §2).

3.1 Promoter dynamics

The changes of the state s of the promoter (described by the first reaction of the system (1)) follows a continuous-time Markov process decribed by the master equation

$$\frac{\mathrm{d}\phi(t)}{\mathrm{d}t} = \mathbf{M}\phi(t) \tag{6}$$

where $\phi(t)$ is the time-dependent probability vector for the promoter state.

This mathematical formalism is commonly used to represent either the stochastic dynamics of a single entity [5, 6] or the deterministic dynamics of a first order rection (eg. metabolic) network[3] [7, 8]. In both cases, it already received extensive mathematical treatment. In particular, equation (6) can be solved from the eigendecomposition of matrix $\mathbf{M}$. We note λ_i (with $i \in [0; 2^N - 1]$) the eigenvalues of $\mathbf{M}$ (stored in the 2^N-vector $\boldsymbol{\lambda}$) and Λ_i its eigenvectors (stored as columns in the $2^N \times 2^N$-matrix $\boldsymbol{\Lambda}$). By construction of matrix $\mathbf{M}$, all the 2^N eigenvalues have a negative real part and one of them (noted λ_0) is null[4]. The corresponding eigenvector Λ_0 (when normalized to 1) is simply the steady-state of the system. Moreover, matrix $\mathbf{M}$ being real, all non-real eigenvalues necessarily come in pairs of conjugates so that the spectrum of $\mathbf{M}$ (the repartition of eigenvalues λ_i on the complex plan) is symmetric.

[2] Note that, for simplicity, the multiplicative Arrhenius constant on $M_{s',s}$ is considered implicitly as an additive constant in the definition of $\hat{E}_{s',s}$.

[3] $\phi(t)$ represent the concentration of all species and $M_{s',s}$ the rate of the reaction that transforms species s in species s'.

[4] More rigorously, there is only one null eigenvalue when the system is ergodic; ie. at least one state is reachable from any other state.

3.1.1 Eigendecomposition of $\mathbf{M}$

In the general case, the eigendecomposition of $\mathbf{M}$ has to be done numerically. But in particular cases of promoter, an analytical expression of eigenvalues λ_i and eigenvectors Λ_i can be obtained:

(i) Two-states promoter: This very simple case ($N = 1$) corresponds to the assumption of most models of stochastic gene expression that consider promoter explicitly [9, 10, 11, 12, 13, 14]. Eigenvalues and eigenvectors are $\lambda_0 = 0, \lambda_1 = -k_{\text{off}} - k_{\text{on}}$ and $\Lambda_0 = [k_{\text{off}}, k_{\text{on}}], \Lambda_1 = [1, -1]$. The steady-state of the system is then $\phi(\infty) = \Lambda_0 / \sum_s \Lambda_{s,0} = [k_{\text{off}}, k_{\text{on}}] / (k_{\text{off}} + k_{\text{on}})$.

(ii) Homogeneous cycle: We consider a promoter which transition graph consists of a cycle of n states (noted $s_0, s_1, \ldots, s_p, \ldots, s_{n-2}, s_{n-1}$ in the sequence of the cycle) where all forward rates are equal (noted k^{f}) as well as all backward rates (noted k^{b}). Outgoing transitions (ie. from a state in the cycle to any other state than the previous and next states in the cycle) are null. Note that off-cycle states are unreachable and can therefore be eliminated for simplicity[5]. This particular system corresponds to the system considered by [15] and its eigendecomposition is:

$$\begin{cases} \lambda_i = k^{\text{f}}(e^{\frac{-2\pi j}{n} i} - 1) + k^{\text{b}}(e^{\frac{2\pi j}{n} i} - 1) \\ \Lambda_{s_p,i} = e^{p \frac{2\pi j}{n} i} \end{cases} \tag{7}$$

(j is the complex unit). This draws on the matrix spectrum a circle (or an ellipse) of n regularly spaced eigenvalues that is tangent to the ordinate axis at 0. It is a regular circle for $k^{\text{b}} = 0$ and flattens as k^{b} increases. It gets flat (ie. all λ_i are real) when $k^{\text{b}} = k^{\text{f}}$.

Exploring numerically how this circle of λ_i changes when transitions are inhomogeneous or when including outgoing transitions indicates that any deviation from the homogeneous, isolated and irreversible cycle results in a flattening of the circle toward the abscissa axis (data not shown).

3.1.2 Autocorrelation and power spectrum of $X(t)$

Once the eigenvalues $\boldsymbol{\lambda}$ and eigenvectors $\boldsymbol{\Lambda}$ are obtained (either analytically or numerically), rewriting $\mathbf{M} = \boldsymbol{\Lambda}\mathbf{D}_\lambda\boldsymbol{\Lambda}^{-1}$ (where $\mathbf{D}_\lambda$ is the diagonal matrix representation of the vector $\boldsymbol{\lambda}$), the integration of the promoter master equation (6) gives:

$$\phi(t + \tau) = \boldsymbol{\Lambda} e^{\mathbf{D}_\lambda \tau} \boldsymbol{\Lambda}^{-1} \phi(t) \tag{8}$$

with $\tau \geq 0$ for now. Considering the promoter in state s_0 at a given instant t_0, the expected transcription rate after a delay τ can be simply deduced from the time evolution of promoter state (8) from $\phi(t_0) = [\delta_{s_0,s}]_{s \in 2^{\mathcal{F}}}$ to $\phi(t_0 + \tau)$. This reads $X(t_0 + \tau) = \sum_s \rho_s \sum_i \Lambda_{s,i} e^{\lambda_i \tau} \Lambda^{-1}_{i,s_0}$. The autocorrelation of process $X(t)$, noted $\tilde{S}_X(\tau) = \langle X(t)X(t+\tau) \rangle$, is the expectation of $\rho_{s_0} X(t_0)$ at steady-state (ie. the sum for all states s_0 weighted by Λ_0). Therefore

$$\tilde{S}_X(\tau) = \sum_{s_0} \rho_{s_0} \Lambda_{s_0,0} \left[\sum_s \rho_s \sum_i \Lambda_{s,i} e^{\lambda_i \tau} \Lambda^{-1}_{i,s_0} \right] \tag{9}$$

Noting that autocorrelations are even functions $\tilde{S}_X(\tau) = \tilde{S}_X(-\tau)$, replacing $e^{\lambda_i \tau}$ by $e^{\lambda_i |\tau|}$ makes the previous equation valid for any $\tau \in \mathbb{R}$. Reordering terms of (9), we find that the autocorrelation of the transcriptional efficiency process is simply written as

$$\begin{cases} \tilde{S}_X(\tau) = \sum_i {\beta_i}^2 e^{\lambda_i |\tau|} \\ \beta_i = \left[\sum_s \rho_s \Lambda_{s,i} \sum_{s'} \Lambda^{-1}_{i,s'} \rho_{s'} \Lambda_{s',0} \right]^{1/2} \end{cases} \tag{10}$$

Note that $\beta_0 = \sum_s \rho_s \Lambda_{s,0} = \langle X \rangle$ is the mean transcription rate.

Taking the Fourier transform of $\tilde{S}_X(\tau)$, remarking that $\int_{-\infty}^{\infty} e^{-\alpha|\tau|} e^{-j\omega\tau} \mathrm{d}\tau = \frac{2\alpha}{\alpha^2 + \omega^2}, \forall \alpha \in \mathbb{C}$, we deduce the power spectrum

$$S_X(\omega) = \sum_i \frac{-2\lambda_i {\beta_i}^2}{{\lambda_i}^2 + \omega^2} \tag{11}$$

[5] If unreachable states are not eliminated, this does not change the expression of eigenvalues we provide but simply results in additional eigenvalues and eigenvectors. These characterize the transitions from off-cycle states to on-cycle states and are not involved when considering a system that starts in an on-cycle state.

3.2 Implications of energy consumption

3.2.1 Boltzmann factor

Thermodynamic approaches can predict the equilibrium steady-state of energy-independent systems. It can be shown simply that, for such systems ($\hat{E}_{s',s} = \hat{E}_{s,s'}$), the steady-state solution Λ_0 of promoter state master equation (6) corresponds to the Bolztmann factor used in these approaches: $\Lambda_{s,0} = \alpha e^{-G_s/\mathrm{k_B}T}$ (with α a normalisation constant). Indeed, it verifies $\mathbf{M}e^{-\mathbf{G}/\mathrm{k_B}T} = \mathbf{0}$

$$\begin{aligned}\sum_s M_{s',s}e^{-G_s/\mathrm{k_B}T} &= \sum_{s\neq s'}\left[e^{-\frac{\hat{E}_{s',s}-G_s}{\mathrm{k_B}T}}e^{-\frac{G_s}{\mathrm{k_B}T}}\right] - \sum_{s''\neq s'}\left[e^{-\frac{\hat{E}_{s'',s'}-G_{s'}}{\mathrm{k_B}T}}e^{-\frac{G_{s'}}{\mathrm{k_B}T}}\right] \\ &= \sum_{s\neq s'}\left[e^{-\frac{\hat{E}_{s',s}}{\mathrm{k_B}T}} - e^{-\frac{\hat{E}_{s,s'}}{\mathrm{k_B}T}}\right] = 0\end{aligned} \tag{12}$$

3.2.2 Energy is necessary (but not sufficient) for periodic activity

A property of interest following the application of this generic Markovian formalism and its energetic reformulation to regulation is that a promoter needs to consume energy for demonstrating a periodic activity. Indeed, this property of Markovian systems can be proved (as in [16] for instance) by showing that when the detailed balance property holds (ie. $M_{s',s}\Lambda_{s,0} = M_{s,s'}\Lambda_{s',0}$), matrix $(\mathbf{D}_{\Lambda_0})^{-1/2}\mathbf{M}(\mathbf{D}_{\Lambda_0})^{1/2}$ is symmetric and hence necessarily have real-valued eigenvalues. Matrices $(\mathbf{D}_{\Lambda_0})^{-1/2}$ and $(\mathbf{D}_{\Lambda_0})^{1/2}$ being diagonal and real-valued, matrix $\mathbf{M}$ also have only real-valued eigenvalues.

So energy-independence prevent any periodic activity (impling that all λ_i are real). However the converse assertion is not true: energy consumption do not necessarily leads to oscillations. For instance, as a counter-example, although matrix $\mathbf{M} = [[-2,1,1,0]^T,[1,-2,0,1]^T,[1,0,-2,1]^T,[0,1,2,-3]^T]$ has a directed cycle and represents a system that effectively consumes energy ($\dot{E} = 3.15\ 10^{-2}\ \mathrm{k_B}T$; cf §3.2.3), all its eigenvalues are real.

3.2.3 Quantification of energy consumption

Although it is possible, for a given system described with the kinetic formulation, to discriminate if it contains energy-dependent transitions or not (by the presence of directed cycles), it is however impossible to determine which of the transitions is so. Conversely, different systems with respect to the energetic formulation can lead to the same kinetic system and hence have exactly the same behavior. Moreover, a given energy-dependent transition can occur more or less frequently in the dynamics of the whole system depending on the other transitions.

However, it is possible to give an expression that quantify concisely energy-dependence for any given system (even formulated kinetically) and that takes into account the dynamics of the system. This is the stationary energy consumption rate[6], noted $\dot{E}$

$$\dot{E} = \mathrm{k_B}T\sum_s \Lambda_{s,0}\sum_{s'\neq s} M_{s',s}\log\frac{M_{s',s}}{M_{s,s'}} \tag{13}$$

3.3 Transmission to RNA and protein levels

We described the stochastic dynamics of molecular complexes at the promoter. Now, we show how these fluctuations transmit through the subsequent steps of gene expression and affect RNA and protein levels.

3.3.1 Inhomogeneous Poisson processes and birth-and-death processes

Both RNA and protein birth-and-death processes are particular cases of the following scheme

$$\mathcal{A} \xrightarrow{\hat{\rho}} \mathcal{A} + \mathcal{B}, \quad \mathcal{B} \xrightarrow{\hat{\gamma}} \varnothing \tag{14}$$

where the level $B(t)$ of $\mathcal{B}$ molecules follows an $\mathcal{A}$-mediated inhomogeneous birth-an-death process.

[6] At steady-state, transition $s \to s'$ occurs at a rate $\Lambda_{s,0}M_{s',s}$ and results in a change of free energy of the system of $\mathrm{k_B}T\log M_{s',s}/M_{s,s'}$ (cf equation (2)). Summing up for all transitions the rate of energy changes provide the stationary energy consumption rate $\dot{E}$ (which is null if the system is energy independent).

Noting the above synthesis and degradation processes $B^+(t)$ and $B^-(t)$ respectively (being sums of Diracs, one at each instant of reaction), we have

$$\mathrm{d}B(t)/\mathrm{d}t = B^+(t) - B^-(t) \tag{15}$$

These two processes being inhomogenous Poisson processes with instantaneous rates $\hat{\rho}A(t)$ and $\hat{\gamma}B(t)$, their power spectra are[7]

$$\begin{cases} S_{B^+}(\omega) = \hat{\rho}^2 S_A(\omega) + \hat{\rho}\langle A\rangle \\ S_{B^-}(\omega) = \hat{\gamma}^2 S_B(\omega) + \hat{\gamma}\langle B\rangle \end{cases} \tag{16}$$

They consist of the spectrum of the time-dependent rate (ie. $S_{\hat{\rho}A(t)}(\omega) = \hat{\rho}^2 S_A(\omega)$) added of a white noise (ie. a *shot noise*) term with a power equal to the mean reaction rate (ie. $\langle B^+\rangle = \hat{\rho}\langle A\rangle$).

Due to dependencies between processes $B^+(t)$ and $B^-(t)$, taking the power spectrum of (15) gives $\omega^2 S_B(\omega) = \hat{\rho}^2 S_A(\omega) + \hat{\rho}\langle A\rangle - \hat{\gamma}^2 S_B(\omega) + \hat{\gamma}\langle B\rangle$. And, because at steady-state $\hat{\rho}\langle A\rangle = \hat{\gamma}\langle B\rangle$, we obtain

$$S_B(\omega) = \overbrace{\underbrace{2\hat{\gamma}\langle B\rangle}_{\text{shot noise}} \;/\; \underbrace{(\hat{\gamma}^2+\omega^2)}_{\text{low-pass filter}}}^{B\text{-intrinsic}} + \overbrace{\underbrace{\hat{\rho}^2 S_A(\omega)}_{\text{rate fluctuation}} \;/\; \underbrace{(\hat{\gamma}^2+\omega^2)}_{\text{low-pass filter}}}^{B\text{-extrinsic}} \tag{17}$$

separating fluctuations comming from within the system (intrinsic) due to stochastic synthesis/degradations of $\mathcal{B}$ molecules and from outside of the system (extrinsic) due variations of concentration of $\mathcal{A}$.

3.3.2 Autocorrelation and power spectrum of $R(t)$ and $P(t)$

Applying this principle to the system (1), we obtain

$$S_R(\omega) = \left[2\gamma\langle R\rangle + \sum_i \frac{-2\lambda_i {\beta_i}^2}{{\lambda_i}^2+\omega^2}\right](\gamma^2+\omega^2)^{-1} \tag{18}$$

$$S_P(\omega) = \left[\overbrace{2\tilde{\gamma}\langle P\rangle}^{\substack{\text{Protein}\\\text{shot noise}}} + \tilde{\rho}^2\left[\overbrace{2\gamma\langle R\rangle}^{\substack{\text{RNA}\\\text{shot noise}}} + \overbrace{\sum_i \frac{-2\lambda_i {\beta_i}^2}{{\lambda_i}^2+\omega^2}}^{\text{Promoter dynamics}}\right]\overbrace{(\gamma^2+\omega^2)^{-1}}^{\substack{\text{Promoter}\to\text{RNA}\\\text{low-pass filter}}}\right]\overbrace{(\tilde{\gamma}^2+\omega^2)^{-1}}^{\substack{\text{RNA}\to\text{protein}\\\text{low-pass filter}}} \tag{19}$$

with $\langle P\rangle = \tilde{\rho}\langle R\rangle/\tilde{\gamma}$ and $\langle R\rangle = \beta_0/\gamma$.

3.3.3 Normalized variances

The mean and the variance of a signal being contained in its power spectrum[8], normalized variances of promoter efficiency, RNA level and protein level (${\sigma_X}^2/\langle X\rangle^2$, ${\sigma_R}^2/\langle R\rangle^2$ and ${\sigma_P}^2/\langle P\rangle^2$ respectively) can be obtained from power spectra (11), (18) and (19):

$$\frac{{\sigma_X}^2}{\langle X\rangle^2} = \sum_{i\neq 0} \frac{{\beta_i}^2}{{\beta_0}^2} \tag{20}$$

$$\frac{{\sigma_R}^2}{\langle R\rangle^2} = \frac{1}{\langle R\rangle} + \sum_{i\neq 0}\left[\frac{{\beta_i}^2}{{\beta_0}^2}\frac{\gamma}{\gamma-\lambda_i}\right] \tag{21}$$

$$\frac{{\sigma_P}^2}{\langle P\rangle^2} = \overbrace{\underbrace{\frac{1}{\langle P\rangle}}_{\text{Poisson}}}^{\substack{\text{from protein}\\\text{low copy number}}} + \overbrace{\underbrace{\frac{1}{\langle R\rangle}}_{\text{Poisson}}\underbrace{\frac{\tilde{\gamma}}{\gamma+\tilde{\gamma}}}_{\substack{\text{RNA}\to\text{prot.}\\\text{time-averaging}}}}^{\substack{\text{from RNA}\\\text{low copy number}}} + \overbrace{\sum_{i\neq 0}\left[\underbrace{\frac{{\beta_i}^2}{{\beta_0}^2}}_{\substack{\text{Molecular}\\\text{interplay}}}\underbrace{\frac{\gamma}{\gamma-\lambda_i}\frac{\tilde{\gamma}}{\gamma+\tilde{\gamma}}\left(1+\frac{\gamma}{\tilde{\gamma}-\lambda_i}\right)}_{\substack{\text{Promoter}\to\text{RNA}\to\text{prot.}\\\text{time-averaging}}}\right]}^{\text{from promoter dynamics}} \tag{22}$$

This generalizes the well-known expression of protein level normalized variance due to [17] that considered a set of N_g independent two-states *on/off* genes. Instanciating our model to this particular system (ie. using case *(i)* in §3.1.1) provides the exact same expression.

[7]For $B^+(t)$ for instance, during a unit of time, there are $\hat{\rho}\langle A\rangle$ events (equivalent to $\hat{\rho}\langle A\rangle$ pairs of events with a null delay) and the expected number of pairs of events separated by a delay between τ and $\tau+\mathrm{d}\tau$ is $\hat{\rho}^2\tilde{S}_A(\tau)\mathrm{d}\tau$. So autocorrelation is $\tilde{S}_{B^+}(\tau) = \hat{\rho}\langle A\rangle\delta(\tau) + \hat{\rho}^2\tilde{S}_A(\tau)$ and its the Fourier transform (Wiener-Khinchin theorem) gives (16).

[8]For any signal $\xi(t)$, $S_\xi(\omega)$ contains the two first moments: $\langle\xi\rangle^2 = \frac{1}{2\pi}\int_{0^-}^{0^+} S_\xi(\omega)\mathrm{d}\omega$ and $\langle\xi^2\rangle = \frac{1}{2\pi}\int_{-\infty}^{+\infty} S_\xi(\omega)\mathrm{d}\omega$

3.3.4 Distribution

The master equation for the system up to the RNA level is

$$\frac{\mathrm{d}\phi_r}{\mathrm{d}t} = \mathbf{D}_{\boldsymbol{\rho}}(\phi_{r-1} - \phi_r) + \gamma((r+1)\phi_{r+1} - r\phi_r) + \mathbf{M}\phi_r \tag{23}$$

where $\mathbf{D}_{\boldsymbol{\rho}}$ is the diagonal matrix representation of $\boldsymbol{\rho}$ and the 2^N-vector $\phi_r(t)$ (time-dependence is implicit in the equation) describes the probability to find the system at a given instant t with r RNA molecules and in the 2^N different promoter states.

The analytical resolution of this master equation is somewhat non-trivial and has only been obtained for a simple two-state system at equilibrium [13, 14]. In the general case, it has to be done numerically. In that purpose, using matrices $\mathbf{M}$, $\mathbf{D}_{\boldsymbol{\rho}}$ and $\mathbf{I}$ (the identity matrix) as blocks of $2^N \times 2^N$, we construct a matrix $\mathbf{M}'$:

$$\mathbf{M}' = \begin{bmatrix} \mathbf{M} & \gamma\mathbf{I} & \mathbf{0} & \mathbf{0} & \mathbf{0} & \dots \\ \mathbf{D}_{\boldsymbol{\rho}} & \mathbf{M} & 2\gamma\mathbf{I} & \mathbf{0} & \mathbf{0} & \dots \\ \mathbf{0} & \mathbf{D}_{\boldsymbol{\rho}} & \mathbf{M} & 3\gamma\mathbf{I} & \mathbf{0} & \dots \\ \mathbf{0} & \mathbf{0} & \mathbf{D}_{\boldsymbol{\rho}} & \mathbf{M} & 4\gamma\mathbf{I} & \dots \\ \mathbf{0} & \mathbf{0} & \mathbf{0} & \mathbf{D}_{\boldsymbol{\rho}} & \mathbf{M} & \dots \\ \vdots & \vdots & \vdots & \ddots & \ddots & \ddots \end{bmatrix} \tag{24}$$

so that the master equation (23) rewrites:

$$\frac{\mathrm{d}}{\mathrm{d}t}[\phi_0, \phi_1, \phi_2, \dots] = \mathbf{M}'[\phi_0, \phi_1, \phi_2, \dots] \tag{25}$$

Although matrix $\mathbf{M}'$ is theoretically of infinite size, its definition can be practically restricted to $r < 2\max(\rho_s)/\gamma$ for instance. Indeed, the tail of the RNA distribution is necessarily upper bounded by the tail of a Poisson distribution of parameter $\max(\rho_s)/\gamma$, therefore stopping the definition of $\mathbf{M}'$ significantly higher than $\max(\rho_s)/\gamma$ guaranties a very negligible inaccuracy

As we are interested in the stationary distribution, we do not need to compute all the eigenvalues and eigenvectors (only the eigenvector with a null eigenvalue provides the stationary distribution). Moreover, matrix $\mathbf{M}'$ being very sparse, its numerical resolution by standard iterative techniques can be very efficient. For instance, obtaining a distribution for the system of figure 4 takes about a second and generating all the data of figure 4 takes only few minutes on a classical laptop computer.

One can apply the same technique for obtaining the distribution of protein level (ie. building a third matrix $\mathbf{M}''$ with $\mathbf{M}'$ as building blocks). But the size of the system makes its resolution practically inefficient in that case and prevents exploration of parameters. This can be overcome by making use of coarsening techniques [18] but this imposes to make strong simplifications on the model (eg. on the birth-and-death processes) that are susceptible to produce inaccuracies.

4 Parameters

Parameter	N	K_A^{d}	K_B^{d}	$[A]$	$[B]$	k_A^{off}	k_B^{off}	k_C^{on}	k_C^{off}
Value	3	10	10	3	3	0.1	0.2	1/30	1/30
Units		nM	nM	nM	nM	s^{-1}	s^{-1}	min^{-1}	min^{-1}

Parameter	ΔG_{AB}	ΔG_{AC}	ΔG_{BC}	γ	$\tilde{\gamma}$	$\tilde{\rho}$	ρ
Value	-2	2	2	1/15	1/30	200 $\tilde{\gamma}$	[2.5, 3, 3, 4, .1, .1, .1, .1]
Units	kcal/mol	kcal/mol	kcal/mol	min^{-1}	min^{-1}	min^{-1}	min^{-1}

Table S1: Parameters for Figure 1 of the main article

Parameter	N	K_A^{d}	K_B^{d}	$[A]$	$[B]$	$1/k_A^{\mathrm{off}}$	$1/k_B^{\mathrm{off}}$
Value	2	0.5	5	$[10^{-2}, 10^{3}]$	5	30	60
Units		nM	nM	nM	nM	s	s

Parameter	ΔE	γ	$\tilde{\gamma}$	$\tilde{\rho}$	ρ
Value	2.5	1/5	1/20	$100\,\tilde{\gamma}$	[0.04, 0.04, 0.4, 0.4]
Units	kcal/mol	min^{-1}	min^{-1}	min^{-1}	min^{-1}

$$\mathbf{k}^0 = \begin{array}{c} \\ A \\ B \end{array} \begin{array}{cccc} \varnothing & A & B & AB \\ \hline a & \bar{a} & a & \bar{a} \\ b & bc & \bar{b} & \bar{b}c \end{array}$$

with $a = \frac{k_A^{\mathrm{off}}}{K_A^{\mathrm{d}}}$, $\bar{a} = k_A^{\mathrm{off}}$, $b = \frac{k_B^{\mathrm{off}}}{K_B^{\mathrm{d}}}$, $\bar{b} = k_B^{\mathrm{off}}$ and $c = e^{-\Delta E/RT}$

Table S2: Parameters for Figure 3 of the main article

Parameter	N	K_A^{d}	K_C^{d}	$[A]$	$[C]$	$1/k_A^{\mathrm{off}}$	$1/k_C^{\mathrm{off}}$	k^{close}
Value	4	20	1	[0.01,100]	[0.01,1000]	20	60	1
Units		nM	nM	nM	nM	s	s	s^{-1}

Parameter	ΔG_{loop}	ΔG_{A-A}	ΔG_{A-C}	$\Delta G_{\mathrm{loop}-A}$	$\Delta E_{\mathrm{loop}-C}$	γ		
Value	9	-2	1.5	-5.5	2.5	1/5		
Units	kcal/mol	kcal/mol	kcal/mol	kcal/mol	kcal/mol	min^{-1}		

Parameter	ρ	
Value	[5,5,10,10, 14,14,14,14, 1,1,3,3, 9,9,9,9]	
Units	min^{-1}	

$$\mathbf{k}^0 = \begin{array}{c} \\ A_1 \\ A_2 \\ C \\ D \end{array} \begin{array}{cccccccccccccccc} \phi & A_1 & A_2 & A_1A_2 & C & A_1C & A_2C & A_1A_2C & D & A_1D & A_2D & A_1A_2D & CD & A_1CD & A_2CD & A_1A_2CD \\ \hline a & \bar{a} & a & \bar{a}g & a & \bar{a} & a & \bar{a}g & a & \bar{a} & a & \bar{a}g & a & \bar{a} & a & \bar{a}g \\ a & a & \bar{a} & \bar{a}g & a & a & \bar{a}h & \bar{a}gh & a & a & \bar{a} & \bar{a}g & a & a & \bar{a}h & \bar{a}gh \\ c & c & c & c & \bar{c} & \bar{c} & \bar{c}h & \bar{c}h & cp & cp & cp & cp & \bar{c}p & \bar{c}p & \bar{c}hp & \bar{c}hp \\ d & d & d & d & d & d & d & d & \bar{d} & \bar{d}l & \bar{d}l & \bar{d}l^2 & \bar{d} & \bar{d}l & \bar{d}l & \bar{d}l^2 \end{array}$$

with $a = \frac{k_A^{\mathrm{off}}}{K_A^{\mathrm{d}}}$, $\bar{a} = k_A^{\mathrm{off}}$, $c = \frac{k_C^{\mathrm{off}}}{K_C^{\mathrm{d}}}$, $\bar{c} = k_C^{\mathrm{off}}$, $d = k^{\mathrm{close}}$, $\bar{d} = k^{\mathrm{close}} e^{\Delta G_{\mathrm{loop}}/RT}$, $g = e^{\Delta G_{A-A}/RT}$, $h = e^{\Delta G_{A-C}/RT}$, $l = e^{\Delta G_{\mathrm{loop}-A}/RT}$ and $p = e^{-\Delta E_{\mathrm{loop}-C}/RT}$

Table S3: Parameters for Figure 4 of the main article

Parameter	N	$[A]$,$[B]$,$[C]$,$[D]$,$[E]$,$[F]$ and $[G]$	t_{slow}	t_{fast}	α
Value	7	3	40	40	0.1
Units		nM	min	s	

Parameter	γ	$\tilde{\gamma}$	$\tilde{\rho}$	ρ
Value	1/10	1/25	$1000\,\tilde{\gamma}$	$6\,\delta_A\,\delta_B\,(1+\delta_C)$
Units	min^{-1}	min^{-1}	min^{-1}	min^{-1}

Table S4: Parameters for Figures 2 and 5 of the main article. NB: The matrix $\mathbf{k}^0$ is obtained from the optimization algorithm described thereafter, using parameters presented in this table. δ_f is the vector of 2^N elements which s-th element is 1 if $f \in s$ and 0 else.

4.1 Optimization algorithm for the eukaryotic promoter example

Matrix $\mathbf{k}^0$ of transition rates is optimized with a Monte Carlo gradient descent algorithm. After a random initialization of matrix $\mathbf{k}^0$ the following is repeated iteratively: 2% of the elements of matrix $\mathbf{k}^0$ are multiplied with independent random variables (ie. reaction energies are added with a normally distributed random number of s.d.=0.3 kcal/mol). This new matrix is accepted and used for the next iteration only if it enhances the following optimization criterion:

$$\frac{\left\langle\left|\dfrac{\mathrm{Im}(\lambda_i)}{\mathrm{Re}(\lambda_i)}\right|\right\rangle\left|\dfrac{\mathrm{Im}(\lambda_1)}{\mathrm{Re}(\lambda_1)}\right|}{\left\langle\left[\frac{t_{\mathrm{fast}}}{t_{\mathrm{slow}}}\frac{\mathrm{Re}(-\lambda_i)}{|\mathrm{Im}(\lambda_1)|/2\pi}-1\right]^2\right\rangle^\alpha}$$

where $\lambda_0, \lambda_1, \ldots, \lambda_{2^N-1}$ are the eigenvalues of $\mathbf{M}$ sorted in increasing order of their modulus (hence $\lambda_0 = 0$). The first term in the numerator promotes for coherent eigenvalues, the second term ensures that the slowest component is the oscillating one and the denominator is a criterion that leads the mean of relaxation times of eigenvalues to be faster than the slow oscillating component with a ratio $t_{\mathrm{fast}}/t_{\mathrm{slow}}$. As depicted on figure 2B in the main article, aperiodic eigenvalues are still widely spread over the real axis and the fastest one have relaxation times as short 6 s.

5 References

[1] A. Sánchez and J. Kondev, "Transcriptional control of noise in gene expression," *Proc. Natl. Acad. Sci. U.S.A.*, vol. 105, pp. 5081–5086, Apr 2008.

[2] G. Ackers, A. Johnson, and M. Shea, "Quantitative model for gene regulation by lambda phage repressor," *Proc. Natl. Acad. Sci. U.S.A.*, vol. 79, pp. 1129–1133, Feb 1982.

[3] L. Saiz and J. Vilar, "Stochastic dynamics of macromolecular-assembly networks," *Mol. Syst. Biol.*, vol. 2, p. 2006.0024, 2006.

[4] L. Bintu, N. Buchler, H. Garcia, U. Gerland, T. Hwa, J. Kondev, and R. Phillips, "Transcriptional regulation by the numbers: models," *Curr. Opin. Genet. Dev.*, vol. 15, pp. 116–124, Apr 2005.

[5] J. Schnakenberg, "Network theory of microscopic and macroscopic behavior of master equation systems," *Rev Mod Phys*, vol. 48, no. 4, pp. 571–585, 1976.

[6] N. van Kampen, *Stochastic processes in physics and chemistry.* New York: North-Holland, 3rd ed., 2007.

[7] J. Z. Hearon, "The kinetics of linear systems with special reference to periodic reactions," *Bull. Math. Biol.*, vol. 15, pp. 121–141, 1953.

[8] D. Summers and J. M. Scott, "Systems of first-orderchemical reactions," *Math. Comput. Modelling*, vol. 10, no. 12, pp. 901–9, 1988.

[9] J. Raser and E. O'Shea, "Control of stochasticity in eukaryotic gene expression," *Science*, vol. 304, pp. 1811–1814, Jun 2004.

[10] I. Golding, J. Paulsson, S. Zawilski, and E. Cox, "Real-time kinetics of gene activity in individual bacteria," *Cell*, vol. 123, pp. 1025–1036, Dec 2005.

[11] B. Kaufmann and A. van Oudenaarden, "Stochastic gene expression: from single molecules to the proteome," *Curr. Opin. Genet. Dev.*, vol. 17, pp. 107–112, Apr 2007.

[12] T. B. Kepler and T. C. Elston, "Stochasticity in transcriptional regulation: Origins, consequences, and mathematical representations," *Biophys J*, vol. 81, no. 6, pp. 3116–36, 2001.

[13] G. Innocentini and J. Hornos, "Modeling stochastic gene expression under repression," *J Math Biol*, vol. 55, pp. 413–431, Sep 2007.

[14] V. Shahrezaei and P. S. Swain, "Analytical distributions for stochastic gene expression.," *Proc. Natl. Acad. Sci. U.S.A*, vol. 105, no. 45, pp. 17256–61, 2008.

[15] V. Lemaire, C. Lee, J. Lei, R. Métivier, and L. Glass, "Sequential recruitment and combinatorial assembling of multiprotein complexes in transcriptional activation," *Phys. Rev. Lett.*, vol. 96, p. 198102, May 2006.

[16] S. Mukherjee, H. Nakanishi, and N. Fuchs, "Markov chain analysis of random walks in disordered media," *Phys Rev E Stat Phys Plasmas Fluids Relat Interdiscip Topics*, vol. 49, pp. 5032–5045, Jun 1994.

[17] J. Paulsson, "Models of stochastic gene expression," *Phys. Life Rev.*, vol. 2, no. 2, pp. 157–75, 2005.

[18] T. Lipniacki, P. Paszek, A. Marciniak-Czochra, A. Brasier, and M. Kimmel, "Transcriptional stochasticity in gene expression," *J. Theor. Biol.*, vol. 238, pp. 348–367, Jan 2006.
